# An App-Based WHO Mental Health Guide for Depression Detection: A Cluster Randomized Clinical Trial

**DOI:** 10.1001/jamanetworkopen.2025.12064

**Published:** 2025-05-23

**Authors:** Brandon A. Kohrt, Akin Ojagbemi, Nagendra P. Luitel, Ioannis Bakolis, Toyin Bello, Paul McCrone, Tatiana Taylor Salisbury, Mark J. D. Jordans, Nicole Votruba, Kenneth Carswell, Eric Green, Evdoxia Gkaintatzi, Bishnu Lamichhane, Olufisayo Elugbadebo, Lola Kola, Heidi Lempp, Neerja Chowdhary, Tarun Dua, Oye Gureje, Graham Thornicroft

**Affiliations:** 1 Center for Global Mental Health Equity, Department of Psychiatry and Behavioral Health, The George Washington University, Washington, DC; 2 World Health Organization Collaborating Centre for Research and Training in Mental Health, Neuroscience, and Substance Abuse, Department of Psychiatry, College of Medicine, University of Ibadan, Ibadan, Nigeria; 3 Research Department, Transcultural Psychosocial Organization Nepal, Kathmandu, Nepal; 4 Centre for Mental Health Policy and Evaluation, Health Service and Population Research Department, Institute of Psychiatry, Psychology and Neuroscience, King’s College London, London, United Kingdom; 5 Institute for Lifecourse Development, University of Greenwich, London, United Kingdom; 6 Centre for Global Mental Health, Health Service and Population Research Department, Institute of Psychology, Psychiatry & Neuroscience, King’s College London, London, United Kingdom; 7 Amsterdam Institute of Social Science Research, University of Amsterdam, Amsterdam, The Netherlands; 8 Nuffield Department of Women’s & Reproductive Health, University of Oxford, Oxford, United Kingdom; 9 Department of Mental Health, Brain Health and Substance Use, World Health Organization, Geneva, Switzerland; 10 Duke Global Health Institute, Duke University, Durham, North Carolina; 11 Centre for Rheumatic Diseases, Department of Inflammation Biology, School of Immunology and Microbial Sciences, Faculty of Life Sciences & Medicine, King’s College London, London, United Kingdom; 12 Department of Psychiatry, Stellenbosch University, Stellenbosch, South Africa

## Abstract

**Importance:**

Depression detection in primary care remains limited in low- and middle-income countries despite increasing use of the World Health Organization Mental Health Gap Action Programme–Intervention Guide (mhGAP-IG).

**Objective:**

To test an app version of the mhGAP-IG (e-mhGAP-IG) in Nepal and Nigeria to improve depression detection.

**Design, Setting, and Participants:**

In this feasibility cluster randomized clinical trial conducted from February 14, 2021, to March 25, 2022, primary care facilities (unit of clustering) in Nepal and Nigeria were randomized to the standard mhGAP-IG training arm (control) or to training using the e-mhGAP-IG app (intervention). Primary care workers (PCWs) received training based on the arm assignment of their health care facility. Statistical analysis was conducted from July 20, 2022, through September 27, 2024.

**Intervention:**

Training using standard mhGAP-IG vs training using the e-mhGAP-IG.

**Main Outcomes and Measures:**

Analysis was performed on an intention-to-treat basis. The main outcome was accuracy of depression detection rates by PCWs, evaluated prior to mhGAP training and 5 to 8 months after training, measured as the percentage of patients who received a depression diagnosis by their PCWs compared with the number of patients who scored 10 or more on the locally validated 9-item Patient Health Questionnaire. Costs per patient detected were calculated.

**Results:**

In Nepal, 25 facilities (67 PCWs; mean [SD] age, 35.3 [9.2] years; 52 men [78%]) were randomized: 13 facilities to standard mhGAP-IG training (36 PCWs) and 12 facilities to e-mhGAP-IG (31 PCWs). In Nigeria, 10 facilities (47 PCWs; mean [SD] age, 46.9 [7.5] years; 44 women [94%]) were randomized: 5 facilities to standard mhGAP-IG (25 PCWs) and 5 facilities to e-mhGAP-IG (22 PCWs). In Nepal, depression detection by PCWs in the standard mhGAP-IG arm increased from 0 of 43 patients before training to 15 of 92 patients after training (adjusted mean change [AMC], 16% [95% CI, 5%-28%]), and depression detection in the e-mhGAP-IG arm increased from 0 of 49 before training to 22 of 91 after training (AMC, 24% [95% CI, 12%-36%]). In Nigeria, depression detection in the standard mhGAP-IG arm increased from 5 of 36 patients before training to 25 of 75 patients after training (AMC, 19% [95% CI, 2%-37%]), and depression detection in the e-mhGAP-IG arm increased from 6 of 35 patients before training to 67 of 76 patients after training (AMC, 71% [95% CI, 57%-85%]). In facilities in the e-mhGAP-IG arm, the app was used for 59 of 616 assessments (10% of patients) in Nepal and 883 of 1077 assessments (82% of patients) in Nigeria. Cost per patient with depression detected using the e-mhGAP-IG was Nepali Rupiya (NPR) 1980 (US $14.79) in Nepal and naira (₦) 1462 (US $0.91) in Nigeria.

**Conclusions and Relevance:**

This feasibility cluster randomized clinical trial demonstrated that the use, cost, and potential clinical benefit of the e-mhGAP-IG varied by setting, highlighting the importance of multisite feasibility studies when evaluating digital innovations intended for health care systems worldwide.

**Trial Registration:**

ClinicalTrials.gov Identifier: NCT04522453

## Key Points

**Question:**

What is the feasibility of using the World Health Organization’s mobile app version of the Mental Health Gap Action Programme–Intervention Guide (e-mhGAP-IG) to improve detection of depression?

**Findings:**

This cluster randomized clinical trial of 35 primary care facilities in Nepal and Nigeria found that in Nepal, the app was used rarely, and depression detection was poor for the standard mhGAP-IG and the e-mhGAP-IG. In Nigeria, the app was commonly used, with 88% of depression cases detected in the group using the e-mhGAP-IG compared with 33% of cases using the standard mhGAP-IG.

**Meaning:**

This study demonstrated the need for feasibility testing of new technologies in multiple settings because adoption may vary and uptake cannot be generalized from a single context.

## Teaser

This cluster randomized clinical trial was conducted in Nepal and Nigeria to test an app version of the World Health Organization (WHO) Mental Health Gap Action Programme–Intervention Guide to improve depression detection.

## Introduction

The World Health Organization (WHO) *Comprehensive Mental Health Action Plan*’s goals for 2030 include delivery of mental health services in primary care in 80% of countries and a 50% increase in service coverage for depression.^1^ One tool to achieve this is the Mental Health Gap Action Programme–Intervention Guide (mhGAP-IG), a training and implementation resource for diagnosis and management of mental health conditions in primary care.^2,3^ The mhGAP-IG is used in more than 90 countries.^4^ However, most primary care workers (PCWs) fail to diagnose depression among their patients; a systematic review found that fewer than 1 in 10 patients with depression are detected in primary care facilities in low- and middle-income countries.^5^

Given the increasing availability of mobile technology to enhance health care,^6,7^ a strategy to improve use of the mhGAP-IG is WHO’s development of an app for use during patient encounters as a decision aid for diagnosing and managing depression and other conditions. This Android-based interactive digital version of mhGAP-IG (e-mhGAP-IG)^8^ has functionality for PCWs to enter patient symptoms and other health information to guide diagnosis and treatment.

The e-mhGAP-IG has potential to improve detection of depression. However, the added investment to implement the app (eg, digital technology infrastructure, training, and supervision) would need to be justified by a clinically relevant level of increased detection. Therefore, a feasibility cluster randomized clinical trial (cRCT) was conducted in Nepal and Nigeria with the goal of determining the potential benefit, app use, and associated costs for improving detection of depression, as well as the feasibility of a future fully powered trial evaluating the e-mhGAP-IG. The study outcomes will provide policymakers, clinicians, and health care managers with crucial information to make informed decisions about implementing the e-mhGAP-IG in their settings.

## Methods

### Design

The full study protocol for this feasibility cRCT has been published^9^ (study protocol in Supplement 1). The current analysis focuses on (1) patient detection outcomes, including e-mhGAP-IG use and cost; (2) PCWs’ depression knowledge, attitudes, and competency for diagnosing and treating depression; and (3) benchmarks for progression to a fully powered trial. Our trial documentation follows the Consolidated Standards of Reporting Trials (CONSORT) reporting guideline, including extensions for pilot and feasibility trials^10^ and cluster trials.^11^ Approval was provided by the Nepal Health Research Council, University of Ibadan/University College Hospital Joint Ethics Committee in Nigeria, King’s College London Research Ethics Committee, and WHO Ethics Review Committee. All PCWs and patients provided written informed consent.

### Setting

The feasibility cRCT was undertaken in 4 phases in Jhapa, Nepal, and Ibadan, Nigeria (eFigure 1 in Supplement 2). Nepal and Nigeria were selected because they were sites of prior studies that evaluated the introduction of mental health services into primary care.^12,13^ Primary care facilities in Jhapa were eligible to participate if they did not have mhGAP-IG training in the prior 24 months. Transcultural Psychosocial Organization Nepal implemented the study. In Nigeria, all regional health facilities in Ibadan were eligible to participate. The study was implemented by the University of Ibadan’s department of psychiatry. In both countries, the primary care services were delivered in government facilities. The trial was conducted in Nepal from February 14, 2021, to March 25, 2022, and in Nigeria from September 1, 2021, to January 31, 2022.

### Participants

#### Primary Care Workers

Within primary care facilities, all PCWs who had prescribing privileges, including PCWs who had prior mental health training, were eligible for mhGAP-IG training. Educational level, years of clinical service, and prior experience delivering mental health care were documented for all participating PCWs.

#### Patients

Patients were recruited in 2 phases: the pretraining phase (for 30 days prior to the PCW mhGAP training) and the patient detection phase (conducted for 90 days during months 5-8 after training). Any patient older than 18 years presenting to the primary care facility who did not have an emergency medical need was eligible.

### Interventions

Training duration in the standard mhGAP-IG arm vs the e-mhGAP-IG arm was time matched, with curricula in both arms lasting 6 days in Nepal and 5 days in Nigeria following existing training practices in the countries. The mhGAP-IG modules for depression, psychosis, alcohol use disorder, and suicide prevention were included in the training. For the e-mhGAP-IG, trainees were given smartphones and introduced to the mobile app on the first day of training in Nepal and on the second day of training in Nigeria. In Nepal, a refresher training on the app was held at 5 months, immediately before the patient detection phase.

Prior to this feasibility study, human-centered design was used in Nepal and Nigeria to refine the e-mhGAP-IG app.^8^ This process resulted in an app with the mhGAP-IG master chart for different conditions, interactive symptom assessment, prompted probing for suicide risk and special populations, and treatment guidance (eFigure 2 in Supplement 2). The app was used on Android phones or tablets. A dashboard that contained information submitted by PCWs through e-mhGAP-IG was available for supervisors.

### Outcomes

#### Patient Outcome

The main patient outcome was accuracy of depression diagnosis, operationalized as the percentage of patients with a 9-item Patient Health Questionnaire (PHQ-9)^14^ score of 10 or more who received a depression diagnosis by a PCW. The PHQ-9 had previously been culturally adapted in Nepali language and clinically validated with primary care patients in southern Nepal; this Nepali PHQ-9 yielded a sensitivity of 94% and specificity of 80% for scores of 10 or more when compared with a structured clinical interview using the Composite International Diagnostic Interview.^15^ In Nigeria, a prior validation study with university students yielded a PHQ-9 score of 10 or more with a sensitivity of 85% and specificity of 99% when compared with the Mini International Neuropsychiatric Interview.^16^

#### PCW Outcomes

Primary care workers’ knowledge was assessed with the mhGAP-IG knowledge assessment tool.^17,18,19^ Attitudes were assessed with the Social Distance Scale (SDS)^20^ and the Revised–Depression Attitudes Questionnaire (R-DAQ).^21^ Competency was evaluated with the WHO-UNICEF (United Nations Children’s Fund) Ensuring Quality in Psychosocial and Mental Health Care competency platform using standardized role plays with the Enhancing Assessment of Common Therapeutic Factors (ENACT) tool^22,23^ (see the eMethods in Supplement 2 for additional information).

### Randomization and Sample Size

Randomization was conducted at the level of the primary care facility. Following best practices for feasibility studies,^24^ there was no between-arm inference testing (ie, no comparison between the standard mhGAP-IG and e-mhGAP-IG arms). Instead, before vs after changes were compared separately within each arm by country. Intraclass correlation coefficients (ICCs) were calculated for baseline measures to estimate variance attributable to clustering. Primary care worker outcomes were visualized using box and whisker plots because of the small sample sizes.

### Health Economic Analyses

In this feasibility exercise, we did not conduct a full economic evaluation. We limited our analyses to estimating the extra cost of the e-mhGAP-IG compared with the mhGAP-IG. We excluded costs of developing the e-mhGAP-IG app. We estimated the cost per patient with depression detected using the standard mhGAP-IG and the extra cost incurred per patient with depression detected when using the e-mhGAP-IG app (the eMethods in Supplement 2 provides additional information on economic analyses).

### Progression to Full Trial

Consistent with best practices in feasibility trials,^25,26^ we established a priori benchmarks for progression to a full trial, as outlined in the study protocol.^9^ The cutoff for PCW retention was completion of all data collection points by at least 67% of PCWs per country. The cutoff for data missingness was no more than 15% per country. The criterion for adverse events was fewer than 10% of patients per country.

### Statistical Analysis

Statistical analysis was conducted using SPSS, version 28 (IBM Corp)^27^ from July 20, 2022, through September 27, 2024. Analysis was performed on an intention-to-treat basis. Following best practices in pilot and feasibility studies, no between-arm hypothesis testing was conducted. For within-arm tests for sensitivity to change, 2-sided tests with *P* < .05 considered significant were used. Generalized estimating equations were used to evaluate within-arm changes in PCW outcomes (knowledge, attitudes, and competency). For depression detection, the outcome was dichotomous (depression detected vs not detected) for each patient with a PHQ-9 score of 10 or more (eMethods in Supplement 2 provides additional information on randomization and sample size calculation for within-arm testing).

## Results

### Cluster and Participant Characteristics

**Figure 1.**
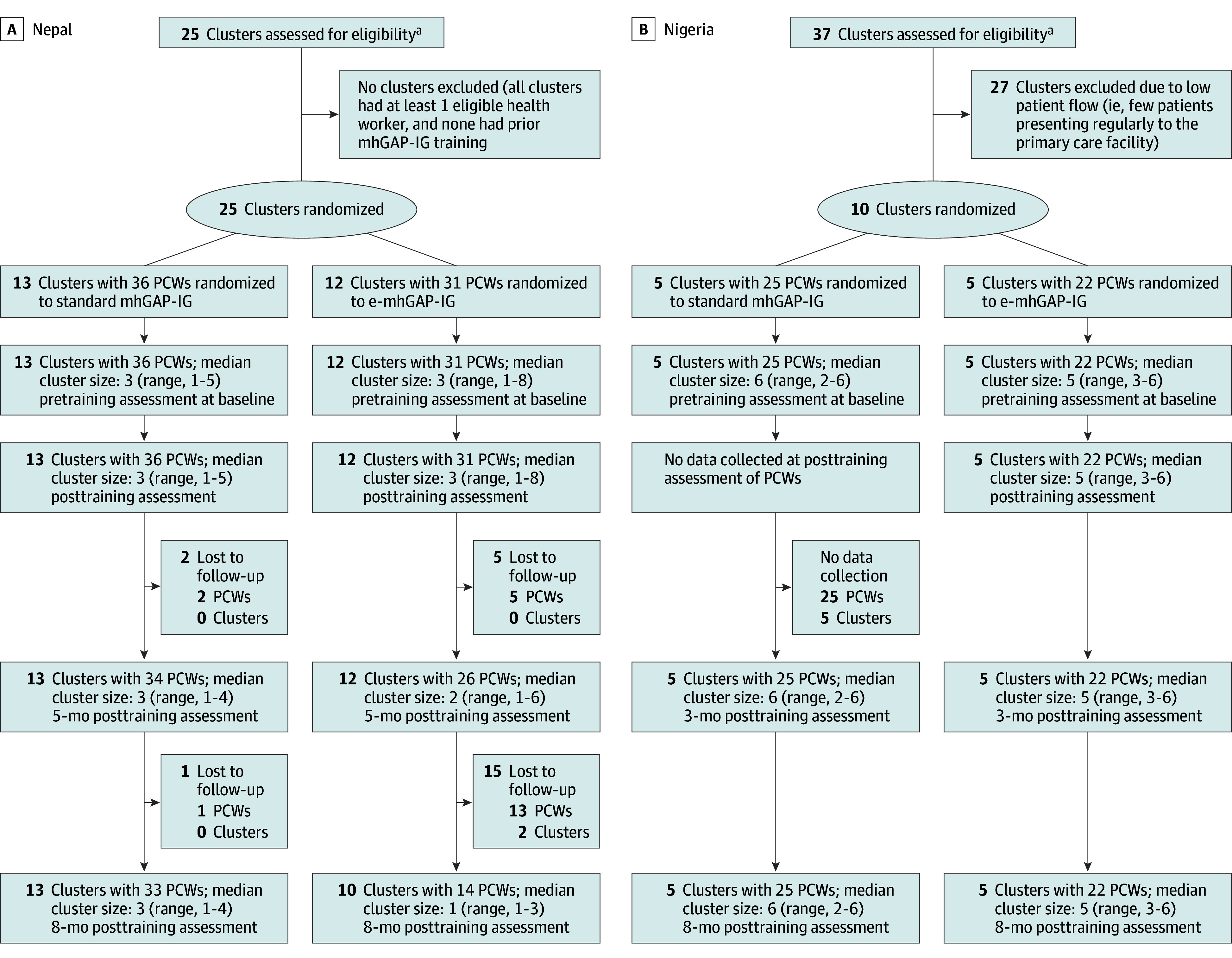
CONSORT Flowchart CONSORT flow diagram for participants in (A) Nepal and (B) Nigeria. e-mhGAP-IG indicates mobile app version of the Mental Health Gap Action Programme–Intervention Guide; mhGAP-IG, Mental Health Gap Action Programme–Intervention Guide; and PCW, primary care worker. ^a^Clusters defined as primary health care facilities.

There were 25 eligible clusters (primary care facilities) in Nepal (Figure 1A). All 25 facilities (67 PCWs; mean [SD] age, 35.3 [9.2] years; 52 men [78%] and 15 women [22%]) were included in the PCW study; 13 primary care facilities were randomized to the standard mhGAP-IG training arm (36 PCWs: 28 men and 8 women), and 12 were randomized to the e-mhGAP-IG arm (31 PCWs: 24 men and 7 women) (Table). Ten of the 25 facilities were included in the patient detection component: 5 facilities from the standard mhGAP-IG arm and 5 facilities from the e-mhGAP-IG arm. In Nigeria, 37 clusters were eligible (Figure 1B), of which 10 facilities (47 PCWs; mean [SD] age, 46.9 [7.5] years; 44 women [94%] and 3 men [6%]) were selected for use in both the PCW and patient detection components: 5 facilities randomized to each arm (standard mhGAP-IG arm, 25 PCWs [24 women and 1 man]; e-mhGAP-IG arm, 22 PCWs [20 women and 2 men]). The Table provides the demographic characteristics of PCWs in the study (see also the eResults in Supplement 2).

**Table.** Demographic Characteristics of Primary Care Workers in Nepal and Nigeria Implementing the mhGAP-IG

Characteristic	No. (%)			
	Nepal (n = 67)		Nigeria (n = 47)	
	Standard mhGAP-IG (n = 36)	e-mhGAP-IG (n = 31)	Standard mhGAP-IG (n = 25)	e-mhGAP-IG (n = 22)
Gender				
Female	8 (22.2)	7 (22.6)	24 (96.0)	20 (90.9)
Male	28 (77.8)	24 (77.4)	1 (4.0)	2 (9.1)
Age, y				
21-35	25 (69.4)	16 (51.6)	0	1 (4.5)
36-50	8 (22.2)	13 (41.9)	13 (52.0)	14 (63.6)
≥51	3 (8.3)	2 (6.5)	12 (48.0)	7 (31.8)
Academic qualification				
Diploma (2 y after secondary school)	14 (38.9)	13 (41.9)	15 (60.0)	9 (40.9)
Bachelor’s degree	14 (38.9)	8 (25.8)	9 (36.0)	10 (45.5)
Master’s degree	3 (8.3)	5 (16.1)	1 (4.0)	0
Other (eg, medical degree)	5 (13.9)	5 (16.1)	0	3 (13.6)
Years working in health care				
<1	3 (8.3)	0	0	0
1-5	11 (30.6)	8 (25.8)	0	0
6-10	11 (30.6)	8 (25.8)	1 (4.0)	1 (4.5)
>10	11 (30.6)	15 (48.4)	24 (96.0)	21(95.5)
Prior mental health training				
No	35 (97.2)	29 (93.5)	0	0
Yes	1 (2.8)	2 (6.5)	25 (100.0)	22 (100.0)

Abbreviations: e-mhGAP-IG, mobile app version of the mental health Gap Action Programme–Intervention Guide; mhGAP-IG, Mental Health Gap Action Programme–Intervention Guide.

In the standard mhGAP-IG arm in Nepal, 246 patients (mean [SD] age, 51 [17.7] years; 131 women [53%] and 115 men [47%]) presented to the primary care facilities during the 1-month pretraining depression detection phase, and 743 patients (mean [SD] age, 48 [16.6] years; 469 women [63%] and 274 men [37%]) presented during the detection phase at 5 to 8 months after training. In the e-mhGAP-IG arm in Nepal, 292 patients (mean [SD] age, 47 [16.5] years; 184 women [63%] and 108 men [37%]) presented during the pretraining detection phase, and 616 patients (mean [SD] age, 49 [17.4] years; 413 women [67%] and 203 men [33%]) presented during the posttraining detection phase. In the standard mhGAP-IG arm in Nigeria, 419 patients (mean [SD] age, 33 [12.4] years; 381 women [91%] and 38 men [9%]) presented during the pretraining detection phase, and 917 patients (864 women, 50 men, and 3 did not report gender; mean [SD] age, 32 [10.5] years; 864 women [94%] and 50 men [6%]) presented during the posttraining detection phase. In the Nigerian e-mhGAP-IG arm, 446 patients (mean [SD] age, 31 [10.9] years; 414 women [93%] and 32 men [7%]) presented during the pretraining detection phase, and 1077 patients (mean [SD] age, 33 [12.8] years; 978 women [91%] and 97 men [9%]) presented during the posttraining detection phase.

### Detection of Depression in Primary Care

**Figure 2.**
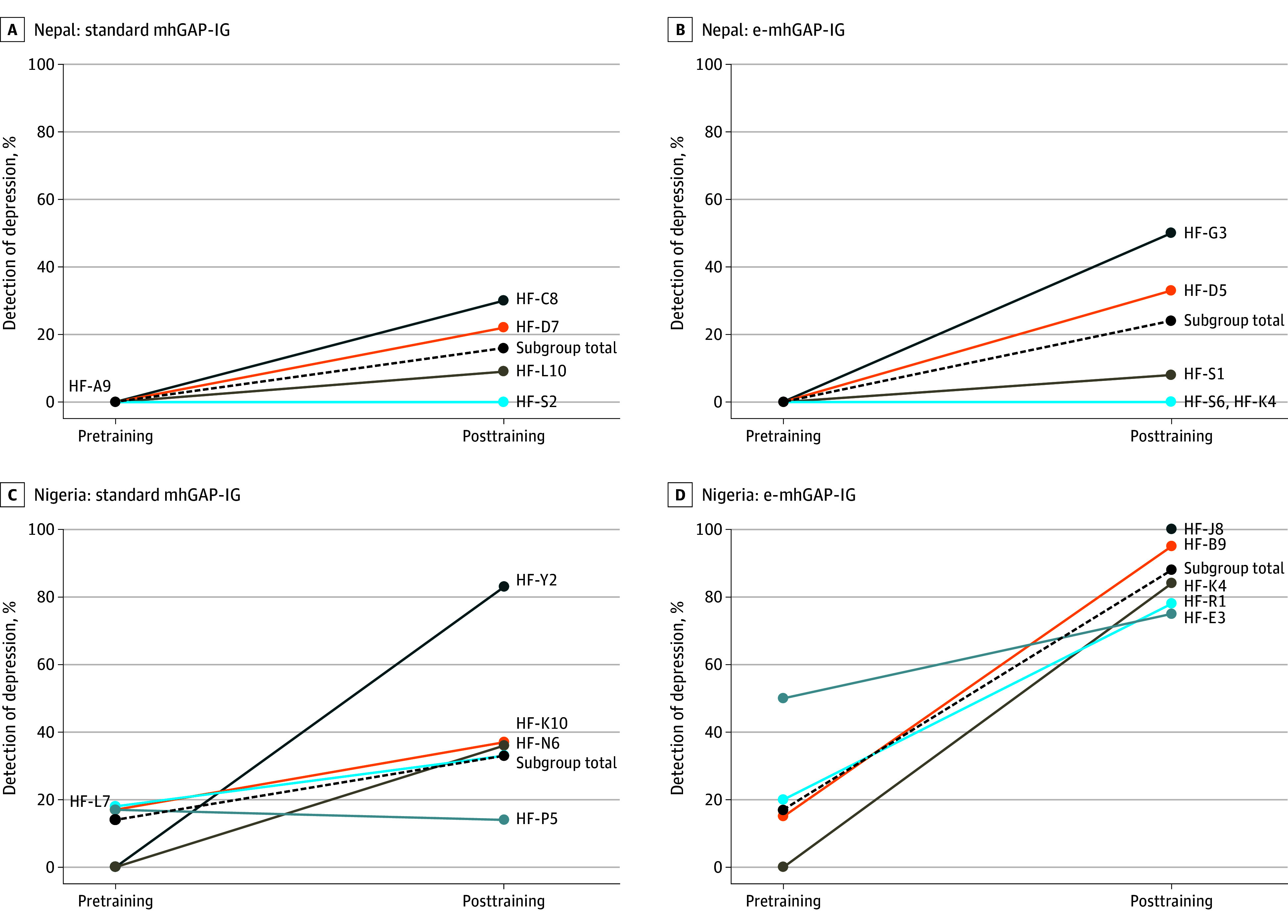
Changes in Detection of Depression Between Pretraining and Posttraining by Country, Study Arm, and Health Facility Percentage detection is based on the number of patients with a 9-item Patient Health Questionnaire (PHQ-9) score of 10 or more who received a diagnosis of depression by the primary care worker. The pretraining detection period was for 1 month prior to training. The posttraining percentage detection period was from 5 to 8 months after the Mental Health Gap Action Programme–Intervention Guide (mhGAP-IG) training. See eFigure 3 in Supplement 2 for additional details on the number of patients presenting per facility, the number of patients with PHQ-9 scores of 10 or more, and rate of app use per facility. e-mhGAP-IG indicates app version of the Mental Health Gap Action Programme–Intervention Guide; HF, health facility code.

During the 1-month pretraining phase in Nepal, none of the patients with PHQ-9 scores of 10 or more received a diagnosis of depression by PCWs in either study arm (ie, 0% detection) (Figure 2; eFigure 3 and eTable 1 in Supplement 2). During the pretraining phase in Nigeria, 5 of 36 patients with PHQ-9 scores of 10 or more received a diagnosis of depression by PCWs in mhGAP-IG arm facilities, and 6 of 35 patients in the e-mhGAP-IG arm received a diagnosis of depression.

During the posttraining patient detection phase in Nepal, PCWs’ diagnoses of patients with depression in the standard mhGAP-IG arm increased from 0 of 43 patients before training to 15 of 92 patients after training (adjusted mean change [AMC], 16% [95% CI, 5%-28%]), and in the e-mhGAP-IG arm, detection increased from 0 of 49 patients before training to 22 of 91 patients after training (AMC, 24% [95% CI, 12%-36%]) (Figure 2; eFigure 3 and eTable 1 in Supplement 2). There was substantial variability across primary care facilities in the e-mhGAP-IG arm in Nepal (ICC, 0.24) compared with an ICC of 0.07 for standard mhGAP-IG facilities.

In Nigeria, detection of depression in the standard mhGAP-IG arm increased from 5 of 36 patients before training to 25 of 75 patients after training (AMC, 19% [95% CI, 2%-37%]) and in the e-mhGAP-IG arm, detection increased from 6 of 35 patients before training to 67 of 76 patients after training (AMC, 71% [95% CI, 57%-85%]) (Figure 2; eFigure 3 and eTable 1 in Supplement 2). With the standard mhGAP-IG, there were not statistically significantly greater odds (odds ratio, 1.13 [95% CI, 0.21-2.05]) of identifying depression after the training compared with detection prior to training. With the e-mhGAP-IG, there were greater odds (odds ratio, 3.58 [95% CI, 2.58-4.59]) of identifying depression after the training compared with before the training. The ICC was 0.14 among standard mhGAP-IG facilities and 0.02 among e-mhGAP-IG facilities.

In Nepal, 14% of depression diagnoses (6 of 43) by PCWs were individuals whose PHQ-9 score was below the cutoff of 10 or more. However, in Nigeria, of 2637 patients scoring below the PHQ-9 score cutoff of 10 or more, none were identified as having depression.

### Use of the e-mhGAP-IG App

In Nepal, the e-mhGAP-IG app was used for 59 of the 616 patients (10%) presenting to the facilities during the patient detection phase (eFigure 4 in Supplement 2). In Nigeria, the app was used for 883 of the 1077 patients (82%). In Nepal, 10 of 15 PCWs never used the app for a patient assessment. The PCWs at the e-mhGAP-IG health facility with the highest detection rate in Nepal never used the app for patient assessments (Health Facility-G3, with depression detected among 18 of 36 patients) (eFigure 3 in Supplement 2). In Nigeria, only 2 of 24 PCWs never used the app for assessments.

### PCWs’ Knowledge, Attitudes, and Competence

**Figure 3.**
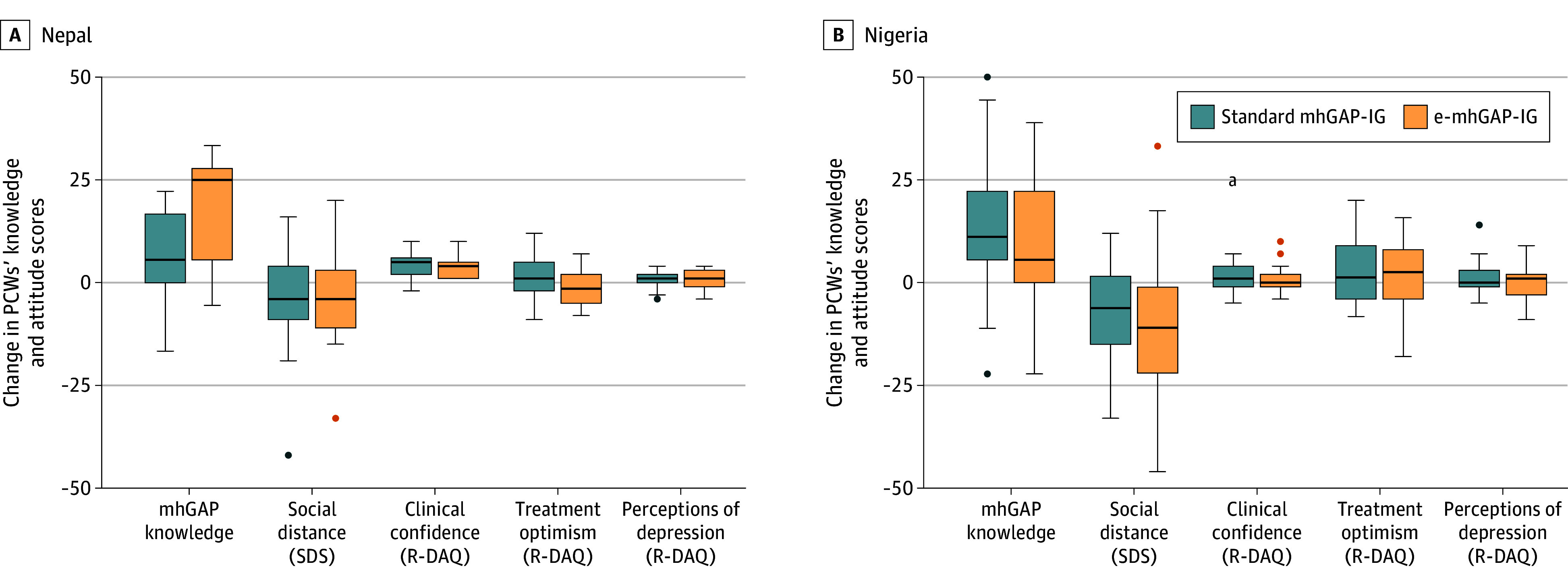
Changes Within Study Arm of Primary Care Worker (PCW) Knowledge and Attitudes Median change and IQR (error bars) for change from before training to 8-month posttraining follow-up assessment. The PCW sample size with data available before training and at 8-month follow-up assessment: Nepal standard mhGAP-IG, 33 PCWs; Nepal e-mhGAP-IG, 14 PCWs; Nigeria standard mhGAP-IG, 25 PCWs; Nigeria e-mhGAP-IG, 22 PCWs. e-mhGAP-IG indicates mobile app version of the Mental Health Gap Action Programme–Intervention Guide; mhGAP-IG, Mental Health Gap Action Programme–Intervention Guide; R-DAQ, Revised–Depression Attitudes Questionnaire; and SDS, Social Distance Scale. ^a^Extreme outlier.

Knowledge of depression increased within arms in all groups (Figure 3; eFigure 5 and eTable 2 in Supplement 2). The change in the SDS score was not significant in either Nepal group but was significant for a reduced SDS score (lower stigma) within both arms in Nigeria. For the R-DAQ, clinical confidence in treating depression improved in both Nepal groups but not in either Nigeria group. Therapeutic optimism (eg, depression improves with treatment) and general perspectives about depression did not show improvement in either country. For clinical competency, both groups in Nepal showed within-arm improvement on the ENACT tool (improved helping skills and reduced harmful behaviors). Clinical competency was not assessed at baseline in Nigeria; therefore, changes could not be evaluated.

### Economic Evaluation

In Nepal, total training costs for both the e-mhGAP-IG and the mhGAP-IG was Nepali Rupiya (NPR) 534 000 (USD $3970). The mean administration time was 30 minutes for the e-mhGAP-IG and 20 minutes for the standard mhGAP-IG. The cost per case of depression detected was NPR 1980 ($14.79) for the e-mhGAP-IG and NPR 2798 ($20.90) for the mhGAP-IG.

In Nigeria, total training costs were naira (₦) 2 480 396 ($1517) for the e-mhGAP-IG and ₦1 233 996 ($750) for standard mhGAP-IG. The mean administration time was 10 minutes for the e-mhGAP-IG and 3 minutes for the mhGAP-IG. The cost per patient detected with depression was ₦1462 ($0.91) for the e-mhGAP-IG and ₦1148 ($0.71) for the standard mhGAP-IG (see the eResults in Supplement 2 for additional details on economic outcomes).

### Progression to Full Trial

With the predetermined cutoff for progression of 67% retention, Nepal met the threshold with 46 of 67 PCWs (69%) completing all 4 assessments. Only 22 of 47 PCWs (47%) in Nigeria completed all 4 assessments. For data missingness, the cutoff was less than 15%. Nepal met the threshold with 10% data missingness (16 092 of 17 956 responses). Nigeria did not, with data missingness at 27% (9171 of 12 596 responses). Both countries were below the cutoff of 10% adverse events among patients. In Nepal, 5 of 1897 patients (0.3%) in the study had suicidality, and in Nigeria, 7 of 2859 patients (0.2%) had suicidality.

## Discussion

This feasibility cRCT sheds light on the potential benefits and challenges of implementing a mobile app version of the mhGAP-IG for use by PCWs to integrate mental health services in primary care. In Nepal, the e-mhGAP-IG was used with only 1 of 10 primary care patients, and both the standard mhGAP-IG and e-mhGAP-IG demonstrated an improvement of only 2 of 10 additional patients with depression detected after training. In Nepal, the PCWs at the facility with the highest depression detection rates did not use the e-mhGAP-IG. In contrast, PCWs in Nigeria used the e-mhGAP-IG with 8 of 10 primary care patients. The PCWs using the e-mhGAP-IG in Nigeria showed improvement, reaching a detection rate of 9 of 10 patients with depression, whereas PCWs using the standard mhGAP-IG detected 3 of 10 primary care patients with depression.

Qualitative research was conducted in both sites as part of the pilot.^28,29^ The qualitative data revealed that in Nigeria, the PCWs were accustomed to using apps on mobile devices for a range of health care services. Apps have been used to provide clinical support information and for documentation (eg, vaccination records). Based on prior experiences with mobile technologies, they reported the e-mhGAP-IG was easy to use. Moreover, the health system enforced the use of health care apps; for example, PCWs were required to keep their mobile phones on at all times and ensure device batteries were charged. Nigerian PCWs also said that using apps in front of patients looked professional, whereas flipping through paper resources looked unprofessional. In Nepal, PCWs reported unfamiliarity with health care apps, and electricity was not available consistently to charge their mobile phones. Familiarity with technology and facility electrical infrastructure likely played a role in country differences in app use. Primary care workers in Nigeria also used the app faster (10 minutes for an e-mhGAP-IG consultation in Nigeria compared with 30 minutes in Nepal). Another difference between countries was prior experience with mental health care; all of the PCWs in Nigeria had prior mental health care experience compared with only 3 of 67 PCWs in Nepal.

### Strengths and Limitations

This study has some strengths; a major strength is the multisite implementation strategy.^30,31^ This study also has some limitations. One methodological limitation was the use of the PHQ-9 to judge the accuracy of diagnosis. The PHQ-9 has high rates of false positives.^32^ Therefore, some individuals with elevated PHQ-9 scores in Nepal and Nigeria may not have met the clinical criteria for depression. For example, a cross-sectional study found that among 1897 primary care patients in Nepal, the prevalence of depression was 15% using a PHQ-9 score cutoff of 10 or more compared with only 6% using the PHQ-9 algorithm for a major depressive episode^33^; this finding suggests that many patients with a PHQ-9 score of 10 or more do not have actual clinical cases of depression. The PHQ-9 also has sensitivity limitations; sensitivity was 94% in Nepal and 85% in Nigeria,^15,16^ suggesting that the PHQ-9 misses some patients with major depressive episodes (ie, false negatives). Although there were some patients who received a diagnosis of depression who scored below the PHQ-9 cutoff, in Nigeria there were no diagnoses of depression made among the 2637 patients who scored below the PHQ-9 cutoff. This highlights a risk of reductionism; the e-mhGAP-IG may oversimplify the depression diagnosis to the administration of the PHQ-9. If a full trial were to be conducted evaluating e-mhGAP-IG, a structured clinical interview (eg, Structured Clinical Interview for the Diagnostic and Statistical Manual of Mental Disorders) could be more accurate than using PHQ-9 as the clinical reference.^34^

Regarding progression to a full trial, Nepal met targets, but in Nigeria, 1 of the 4 assessments in the standard mhGAP-IG arm was not completed, and competency evaluations were not completed at baseline. This was partly due to research procedures being disrupted during the COVID-19 pandemic. If proceeding to a full trial, it would help to develop mechanisms to ensure completion at all study time points of all measures.

## Conclusions

This feasibility cRCT of the e-mhGAP-IG app in Nepal and Nigeria provides valuable insights into the potential of digital tools to enhance depression detection in primary care in low- and middle-income countries. Evaluating new interventions and implementation strategies in multiple sites is recommended for global mental health studies given differences in culture, health systems, technological literacy, and help-seeking for mental health conditions.^30,31^ If this study had been conducted only at 1 site, the feasibility and potential benefit of the e-mhGAP would have been overestimated or underestimated, and the major influence of context on implementation would not have been evident.^30^ As primary care services strive to meet 2030 WHO mental health targets, thoughtful evaluation of digital tools, such as the e-mhGAP-IG, combined with robust training and supervision and a supportive technological infrastructure can play a crucial role in expanding access to quality mental health services worldwide.

## Notes

Supplement 1.
Study Protocol

Supplement 2. **eMethods.** Additional Information **eFigure 1.** Phases of the Feasibility Cluster Randomized Controlled Trial **eFigure 2.** Screenshots of e-mhGAP-IG App for Android Operating System **eResults.** Additional Information **eFigure 3.** Changes by Health Facility in Detection of Depression by Primary Care Workers in the Standard Mental Health Gap Action Programme–Intervention Guide Arm and Mobile App Version of the mhGAP-IG Arm **eTable 1.** Depression Detection by Facility and Country **eFigure 4.** App Usage by Country and Provider **eFigure 5a.** Median Scores With Interquartile Range for mhGAP Knowledge for Primary Care Providers **eFigure 5b.** Median Scores With Interquartile Range for Social Distance Scale (SDS) for Primary Care Providers **eFigure 5c.** Median Scores With Interquartile Range for Clinical Confidence (Revised Depression Attitudes Questionnaire, R-DAQ, Domain 1) for Primary Care Providers **eFigure 5d.** Median scores With Interquartile Range for Treatment Optimism (Revised Depression Attitudes Questionnaire, R-DAQ, Domain 2) for Primary Care Providers **eFigure 5e.** Median Scores With Interquartile Range for Perception of Depression (Revised Depression Attitudes Questionnaire, R-DAQ, Domain 3) for Primary Care Providers **eFigure 5f.** Median Scores With Interquartile Range for Helpful Behaviors on Clinical Competencies (Enhancing Assessment of Common Therapeutic Factors, ENACT) for Primary Care Providers **eFigure 5g.** Median Scores With Interquartile Range for Harmful Behaviors on Clinical Competencies (Enhancing Assessment of Common Therapeutic Factors, ENACT) for Primary Care Providers **eTable 2.** Means and 95% Confidence Intervals for Changes From Pre-Training to 8-Month Follow-Up Among Primary Care Providers‘ Knowledge, Attitudes, and Clinical Competence Adjusted for Clustering and Repeated Measures
eReferences.

Supplement 3.
Data Sharing Statement
