# Supplementary material for: An App-Based WHO Mental Health Guide for Depression Detection: A Cluster Randomized Clinical Trial
Source: JAMA Netw Open. 2025 May 23;8(5):e2512064. doi: 10.1001/jamanetworkopen.2025.12064 (PMC12102703; doi:10.1001/jamanetworkopen.2025.12064)
Supplement: Supplement 1. — Study Protocol [file jamanetwopen-e2512064-s001.pdf]

Study Protocol: Adaptation of the World Health Organization Electronic Mental Health Gap Action Programme Intervention Guide App for Mobile Devices in Nepal and Nigeria: Protocol for a Feasibility Cluster Randomized Controlled Trial

Abstract

Background

There is a growing global need for scalable approaches to training and supervising primary care workers (PCWs) to deliver mental health services. Over the past decade, the World Health Organization Mental Health Gap Action Programme Intervention Guide (mhGAP-IG) and associated training and implementation guidance have been disseminated to more than 100 countries. On the basis of the opportunities provided by mobile technology, an updated electronic Mental Health Gap Action Programme Intervention Guide (e-mhGAP-IG) is now being developed along with a clinical dashboard and guidance for the use of mobile technology in supervision.

Objective

This study aims to assess the feasibility, acceptability, adoption, and other implementation parameters of the e-mhGAP-IG for diagnosis and management of depression in 2 lower-middle-income countries (Nepal and Nigeria) and to conduct a feasibility cluster randomized controlled trial (cRCT) to evaluate trial procedures for a subsequent fully powered trial comparing the clinical effectiveness and cost-effectiveness of the e-mhGAP-IG and remote supervision with standard mhGAP-IG implementation.

Methods

A feasibility cRCT will be conducted in Nepal and Nigeria to evaluate the feasibility of the e-mhGAP-IG for use in depression diagnosis and treatment. In each country, an estimated 20 primary health clinics (PHCs) in Nepal and 6 PHCs in Nigeria will be randomized to have their staff trained in e-mhGAP-IG or the paper version of mhGAP-IG v2.0. The PHC will be the unit of clustering. All PCWs within a facility will receive the same training (e-mhGAP-IG vs paper mhGAP-IG). Approximately 2-5 PCWs, depending on staffing, will be recruited per clinic (estimated 20 health workers per arm in Nepal and 15 per arm in Nigeria). The primary outcomes of interest will be the feasibility and acceptability of training, supervision, and care delivery using the e-mhGAP-IG. Secondary implementation outcomes include the adoption of the e-mhGAP-IG and feasibility of trial procedures. The secondary intervention outcome—and the primary outcome for a subsequent fully powered trial—will be the accurate identification of depression by PCWs. Detection rates before and after training will be compared in each arm.

Results

To date, qualitative formative work has been conducted at both sites to prepare for the pilot feasibility cRCT, and the e-mhGAP-IG and remote supervision guidelines have been developed.

Conclusions

The incorporation of mobile digital technology has the potential to improve the scalability of mental health services in primary care and enhance the quality and accuracy of care.

Trial Registration

ClinicalTrials.gov NCT04522453; https://clinicaltrials.gov/ct2/show/NCT04522453.

International Registered Report Identifier (IRRID)

PRR1-10.2196/24115

Keywords: mental health, community mental health, digital technology, primary health care, intervention, eHealth, mHealth, LMIC, remote supervision, training, mobile phone

Introduction

Background

Mental illnesses are common, affecting 1 in every 3 people during their lifetime [1]. Globally, mental illnesses are the leading contributor of years lived with a disability [2]. Despite the prevalence and impact of mental illness, a large difference between true and treated prevalence rates of mental disorders, also known as the mental health treatment gap, exists. It is estimated that more than 80% of people with severe mental illness in low- and middle-income countries (LMICs) receive no treatment [3]. Only 16.5% of people with depression living in LMICs have access to minimally adequate treatment [4]. The consequences of this treatment gap include symptom persistence and deterioration, social exclusion, and long-term disability of people who could be economically productive and socially included. Globally, there is growing recognition of the importance of mental health, as evidenced by its incorporation in the United Nations 2030 Agenda for Sustainable Development and extension of the World Health Organization (WHO) Comprehensive Mental Health Action Plan to 2030 by the World Health Assembly [5].

The limited number of mental health specialists and the concentration of care in hospital settings in urban rather than rural areas limit the availability and accessibility of care [6]. Low treatment rates in LMICs are related to poor demand and supply-side forces. High levels of stigma associated with mental illness manifest in low rates of help seeking among those who would benefit from care [7-10]. The WHO recommends a task-shifting approach to strengthen the generalist workforce and improve access to health care, including mental health care [11]. However, this method requires the availability of evidence-based tools and appropriate training, supervision, and support.

In recent years, there has been an exponential rise in global access to mobile technologies in LMICs. In 2012, there were 287 million unique mobile phone subscribers across sub-Saharan Africa, covering 32% of the population [12]. Moreover, 6 years later, that number rose to 465 million, representing 44% of the population. In Nepal, the number of mobile contracts (27.85 million) surpasses the total population (26.49 million) [13]. The increased application of mobile technology to the health care arena, known as mobile health (mHealth), aims to provide a powerful platform to improve the quality of interventions using a task-shifting approach and reduce the treatment gap. mHealth refers to the use of mobile technology in health interventions and service provision [14]. In a recent WHO survey, 87% of the responding countries reported at least one government-sponsored mHealth program in their country [14]. However, only 14% of countries reported an evaluation of these programs, raising concerns about insufficient evidence of impact.

A systematic review of smartphone use in clinical decision-making by health care professionals identified 7 randomized controlled trials conducted in high-income settings, which demonstrated improved knowledge, diagnosis, treatment decisions, and documentation using mHealth technology [15]. Studies on mHealth tools in LMICs have yielded mixed results [16]. Qualitative data, however, suggest that the intervention facilitated task shifting and improved health workers' morale.

In 2010, the WHO launched the Mental Health Gap Action Programme Intervention Guide (mhGAP-IG) [17], an evidence-based assessment and management guide for mental, neurological, and substance use conditions designed for use by primary and community health staff in LMICs [18].

The first edition of the mhGAP-IG (v1.0) has been implemented in over 100 countries. An updated version (v2.0) was launched in 2016, with new sections and updated evidence-based guidance [17], along with a first version of a smartphone app available for both Android and iOS devices in 2017. The mhGAP-IG v2.0 consists of 8 modules addressing priority conditions (ie, depression, psychoses, epilepsy, child and adolescent mental and behavioral disorders, dementia, disorders due to substance use, self-harm or suicide, and other significant mental health complaints that impair daily functioning or lead to help seeking). It provides an overview of common presentations for each condition, followed by detailed guidance for assessment, management (including referral to specialist care), and follow-up.

The Emilia (E-mhGAP Intervention Guide in Low- and Middle-Income Countries: Proof-of-Concept for Impact and Acceptability) project seeks to readdress the treatment gap by developing a potentially practical way for primary care workers (PCWs) to diagnose and treat people with mental illness according to evidence-based guidelines.

Aims and Objectives

Emilia aims to test the feasibility of an updated electronic Mental Health Gap Action Programme Intervention Guide (e-mhGAP-IG) and trial procedures for the future conduct of a large-scale trial, which would evaluate differences in depression detection between facilities using the e-mhGAP-IG versus the paper mhGAP-IG. The objectives of this feasibility study, in preparation for a future trial, include the following:

1. To evaluate the feasibility and overall implementability of primary care mental health services using the e-mhGAP-IG for training, supervision, and delivery of care (primary objective).
2. To determine recruitment and retention rates of PCWs and patients.
3. To establish the acceptability and feasibility of assessing PCW and patient outcomes.
4. To assess ethics and safety procedures using adverse event reporting.
5. To describe depression detection rates in primary health clinics (PHCs).
6. To describe depression treatment outcomes in PHCs.

Methods

Settings

The study will take place within the administrative districts of Nepal (Jhapa administrative district) and Nigeria (Ibadan North, Ibadan North West, Ona Ara, and Akinyele local government areas). In each country, a minimum of 6 PHCs will be recruited for the study to represent a range of urban and rural settings.

Nepal is classified as a lower-middle–income country, with an estimated population of 28.1 million [19]. In a recent survey, 16.8% of individuals attending primary care facilities met the criteria for depression [20]. However, only 8.1% had sought care for their mental health [21]. Primary care facilities include PHCs, health posts, urban health centers, community health units, and primary

health care outreach clinics. In these facilities, services are delivered by medical officers, health assistants, staff nurses, auxiliary health workers, and auxiliary nurse midwives. However, medical officers and staff nurses are only available in PHCs [22]. The availability of mental health care in the country is limited to services largely provided through hospitals located in the larger cities [22]. The Jhapa administrative district has a total population of 812,650 people. Medical care is provided through 1 zonal hospital, 6 PHCs, 44 health posts, and 6 urban health centers [23]. Specialist outpatient mental health services are located in 2 private hospitals within the district. The mhGAP-IG was adopted by the government of Nepal and implemented in several districts after the major earthquake in 2015. The government has allocated a budget for district-level mental health care services to include the addition of 6 psychotropic drugs to those already freely available within health facilities and the strengthening of community mental health care services [24].

Nigeria is classified as a lower-middle–income country. It is home to the largest national population in Africa (195.9 million as of 2018) [25]. Recent research in the country estimated a 5.5% prevalence of depression [26]. However, similar to other LMICs, 85% of individuals living with a mental disorder receive no treatment [27]. Ibadan metropolis has 11 local government areas and a population of approximately 3.5 million people. The mental health services in Ibadan are primarily provided by 2 large general hospitals. There are 186 PHCs, each serving a population of approximately 10,000 people. The study will be conducted in 2 urban and 2 rural local government areas. PHCs in Nigeria are staffed by nonphysician health workers (nurses, community health officers, and community health extension workers) who provide treatment for common disorders (including depression) presenting in primary care. The country adopted the mhGAP-IG as a national program for expanding mental health services in 2013, and PHCs are among those where providers have received training in the use of the mhGAP-IG. In Ibadan, PCWs are provided with unstructured supervision by a supervisory general practitioner who typically oversees a group of 6-8 PHCs within a local government area.

The use of digital technology in both Nepal and Nigeria has seen exponential growth in recent years, with the trend expected to continue. In Nepal, mobile penetration was 133% in 2018, which is greater than 100% because most Nepalis have multiple mobile phone provider contracts [28]. In the same year, mobile penetration was estimated to be 49% in Nigeria, with a projected increase to 55% by 2025 [29]. A total of 36% of mobile phone connections in Nigeria are linked to a smartphone.

Technology

A 2015 WHO consultation on the 5-year impact of the mhGAP-IG v1.0 highlighted the demand for an e-version. Respondents identified increased utility and coverage of an electronic guide as reasons for its development. An e-version also creates new opportunities for quality improvement (eg, in remote supervision). A year later, a privately developed e-version of the mhGAP-IG for use in Afghanistan was used for 3000 screenings and 600 referrals [30]. Community health workers reported good acceptability of the mobile app. An e-version of the mhGAP-IG v2.0 was launched by the WHO in October 2017 for Apple and Android smartphones and tablets.

The Emilia project comprises 3 phases: (1) development of an adapted e-mhGAP-IG, (2) feasibility testing, and (3) knowledge transfer and future work. In phase 1, an updated version of the WHO's electronic intervention guide was developed using a human-centered design approach. Human-

centered design is an approach that actively engages stakeholders in the design process using cutting-edge methods to ensure that interventions are optimized for both front-line use and local and national implementation [31]. The approach includes qualitative research with key stakeholders to identify motivations, an iterative process of intervention development and prototyping, and intervention evaluation. The updating of the e-mhGAP-IG included individual and group interviews with PCWs in Nepal and Nigeria to understand the accessibility of technology by health workers, their use patterns, and their preferences for the design of the e-mhGAP-IG. The findings showed that most health workers had access to a personal smartphone, were familiar with the use of various smartphone apps, and valued the idea of an e-version of the mhGAP-IG. Requested features included decision support functions, ability to be used in offline mode, and an easy-to-use design that limited text entry. The formative work also highlighted the importance of recording patient information for review and use in supervision through a clinical dashboard. The resulting updated app features a *reference mode* for training and exploration and a *patient mode* for completing an assessment or management visit with an individual. Health workers can access a brief description of possible conditions through the *Master Chart* (a feature of mhGAP-IG 2.0) and then select modules for further assessment. The mhGAP-IG algorithms are presented in a single-page series of yes or no questions with a *proceed* button, indicating when the end of an algorithm has been reached. Health workers are then presented with an assessment summary, including any additional information to consider, such as whether the individual belongs to a *special population* that may affect treatment decisions. Within the app, health workers can complete additional information for a patient's record, including measures of severity, functioning, and information for follow-up visits. All information is accessible in a clinical dashboard that summarizes key information for each visit as well as aggregate information to help supervisors identify any issues that can be addressed in supervision (eg, overmedication or inaccurate diagnosis). The e-mhGAP-IG will be available in both English and Nepali. Prototypes of the app have been tested with health workers in Nepal (5 iterations) and Nigeria (4 iterations) who found the app to have an intuitive design that is appropriate and feasible for use in clinical work.

Study Design

A feasibility cluster randomized controlled trial (cRCT) will be conducted to evaluate and compare the implementation outcomes [32] and clinical outcomes of the adapted e-mhGAP-IG v2.0 and the paper version of the mhGAP-IG v2.0. A total of 10 PHCs in each country (Nepal and Nigeria) will be randomized to training, supervision, and care delivery using the paper mhGAP-IG (control arm) or the e-mhGAP-IG (experimental arm), and outcomes of PCWs and patients will be collected over a 9-month period (Figure 1). Data collected through the feasibility study will be used to further refine the intervention (eg, acceptability and feasibility for randomization and recruitment) and power a subsequent cRCT.

Figure 1.

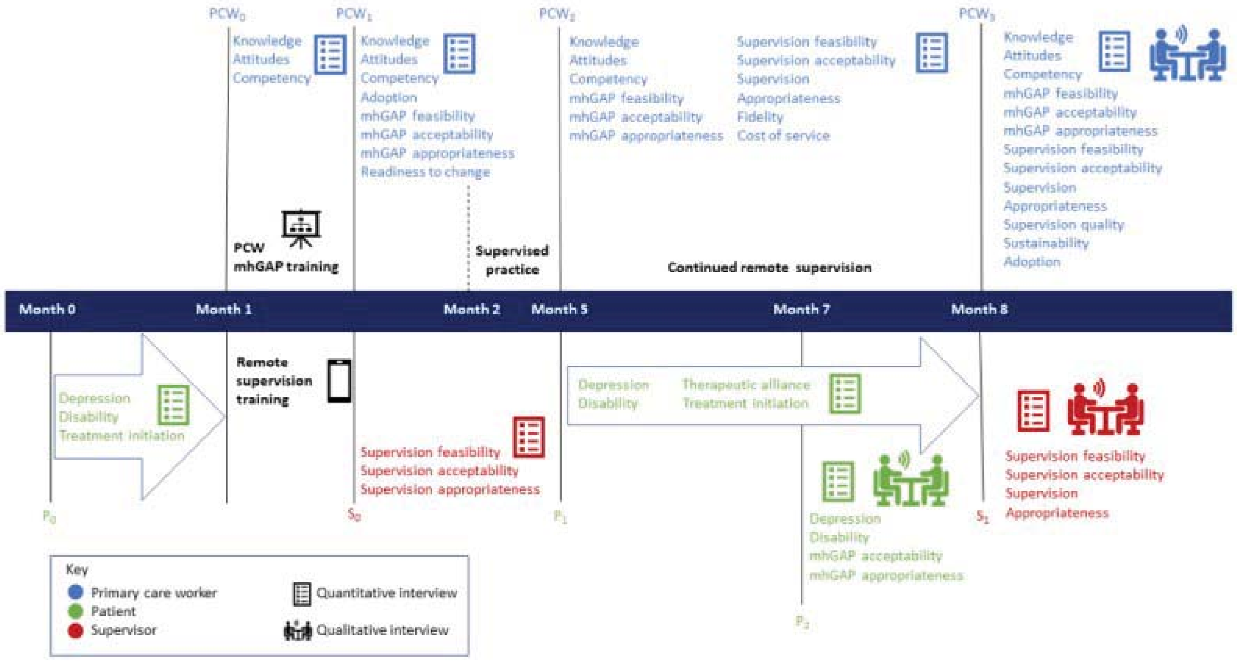

Feasibility study patient and provider data collection procedure. mhGAP: Mental Health Gap Action Programme; P_0: patient pre training assessment; P_1: patient baseline health care appointment assessment; P_2: patient 3 months post baseline health care appointment; PCW: primary care worker; PCW_0: primary care worker baseline assessment; PCW_1: primary care worker immediate post training assessment; PCW_2: primary care worker 3 months post training assessment; PCW_3: primary care worker 8 months post training assessment; S_0: Supervisor baseline assessment; S_1: Supervisor 8 months post training assessment.

Participants

An estimated 20 PHCs in Nepal and 6 PHCs in Nigeria will be identified by local partners. Clinic managers and PCWs will be approached by local research staff with invitations to participate in the study. On the basis of the staffing of PHCs, we estimate that 2-5 PCWs per PHC will participate, equivalent to 40 PCWs in Nepal and 30 PCWs in Nigeria, approximately 15-20 health workers per arm per country (Figure 2).

Figure 2.

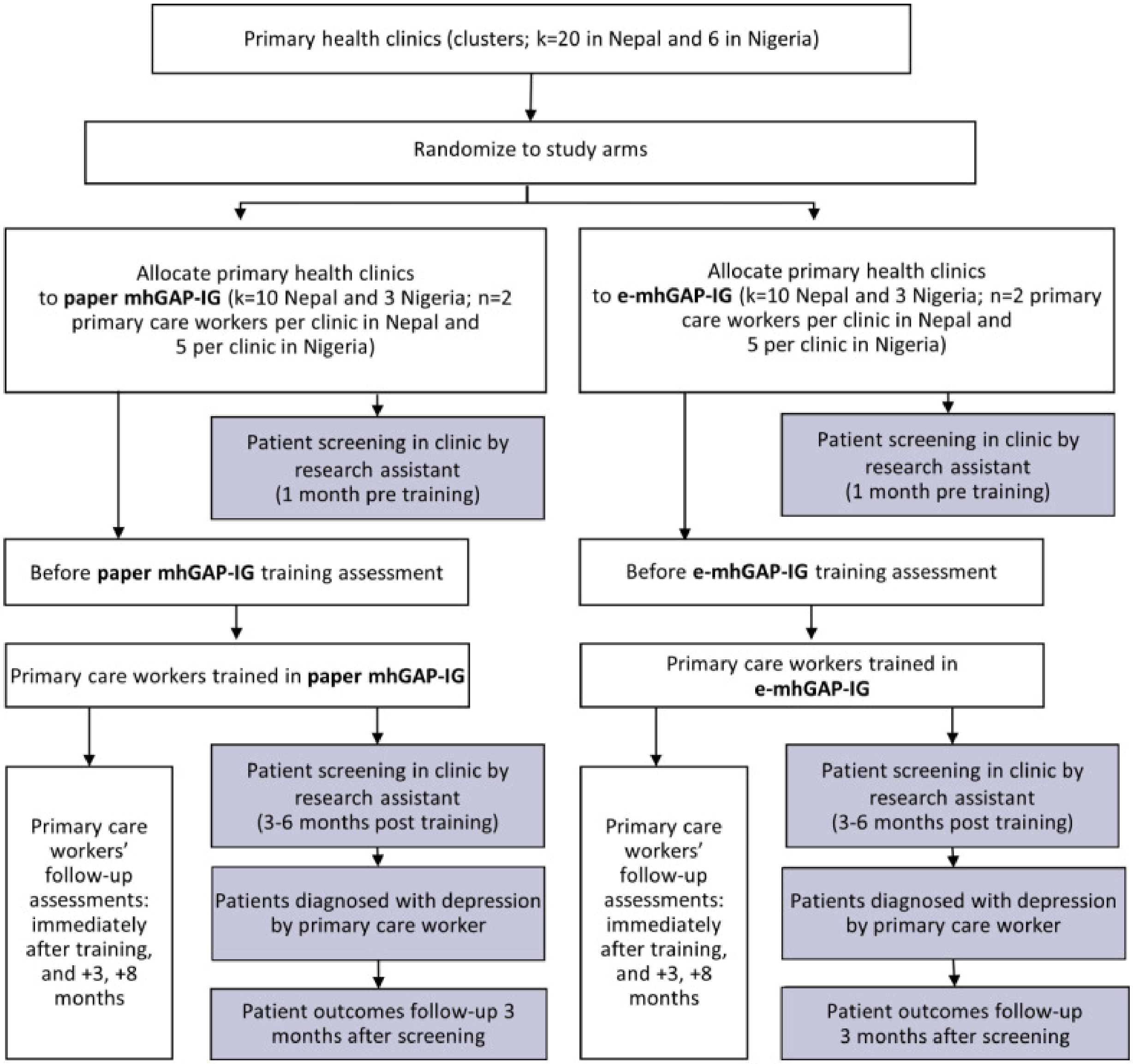

Emilia (E-mhGAP Intervention Guide in Low- and Middle-Income Countries: Proof-of-Concept for Impact and Acceptability) flow chart. e-mhGAP-IG: electronic Mental Health Gap Action Programme Intervention Guide; mhGAP-IG: Mental Health Gap Action Programme Intervention Guide.

PCWs will be eligible to participate if they are employed by the PHC or government and have roles and responsibilities related to the use of the mhGAP-IG (eg, direct clinical use or supervision). All relevant PCWs, regardless of individual study participation, will receive training in the mhGAP-IG v2.0 (electronic or paper version) and have ongoing remote support and supervision by health care providers with enhanced mental health knowledge.

Patients presenting to primary care will be enrolled in the study to evaluate their perceptions of services and to obtain descriptive information on detection rates and treatment impact to inform a

subsequent fully powered cRCT. There will be 2 periods of patient enrollment: *pre training*, which is before PCWs receive mhGAP-IG training, and *post training*, which is after PCWs have received mhGAP-IG training. For the 1-month before the mhGAP-IG training, a random selection of adults presenting to primary care will be screened by research assistants to determine depression status, which will be compared with documented diagnoses by the PCWs. Similarly, beginning at 3 months post training, a random selection of patients will be screened by research assistants for depression status. This posttraining patient enrollment will last a minimum of 3 months.

On the basis of prior data on primary care service use and screening depression rates, we anticipate being able to screen approximately 50% of adult patients presenting to primary care, with a possibility of screening a higher percentage depending on patient flow in the facility [33]. Therefore, we anticipate screening approximately 200 patients per arm per country per month (ie, 400 patients per country in the 1-month pretraining patient enrollment period and 1200 patients per country in the 3-month posttraining patient enrollment period).

Inclusion criteria for patients to be screened include the following:

- Attending PHC for treatment of a new case at recruitment
- Reached the age of adulthood (ie, ≥18 years)
- Fluent in Nepali (Nepal only) or English or Yoruba (Nigeria only).

Adult attendees will be deemed ineligible for the study if they are unable to understand or complete study assessment (eg, individuals with severe learning disability or dementia), unable to provide informed consent, or have a medical emergency requiring immediate intervention.

All patients who meet the eligibility criteria and choose to enroll in the study will then be screened by a research assistant to determine their depression status. Following this screening, patients will be evaluated by a PCW who will make an independent diagnosis without speaking to the research assistant or reviewing the screening results. Administration of screening tools may occur before or after the patient meets the health worker based on the workflow of the clinics. After patients meet the PCWs, a research assistant will review the provider's case notes to document whether or not a depression diagnosis was made.

Study Arms

Intervention Condition

The intervention will consist of (1) availability of the adapted e-mhGAP-IG for use on a clinic tablet, (2) participation in remote supervision and the support module developed in phase 1 for implementation in Nepal and Nigeria, and (3) training to all relevant PCWs and supervisors in the use of the adapted e-mhGAP-IG and clinical dashboard. Health care providers with enhanced mental health knowledge who have attended the training of trainers workshops for the e-mhGAP-IG and remote supervision will act as supervisors for the purpose of the study. These might include, but are not limited to, psychiatrists, mental health nurses, general physicians, senior PCWs, and counselors.

Control Condition

The control condition will consist of (1) availability of the paper version of the mhGAP-IG v2.0 adapted for use in Nepal and Nigeria, (2) participation in remote supervision and the support module developed in phase 1 for implementation in Nepal and Nigeria, and (3) training to all relevant PCWs and supervisors in the use of the paper version of the mhGAP-IG v2.0 and supervision from distance (Table 1). Trained specialists in the research teams within each study site will act as supervisors for the purpose of the study. Supervisors are intended to be health care providers with enhanced mental health knowledge who have attended the training of trainers workshops for the paper mhGAP-IG v2.0. These might include, but are not limited to, psychiatrists, mental health nurses, general physicians, senior PCWs, and counselors.

Table 1.

Feasibility study arm components.

Component	Intervention condition	Control condition
e-mhGAP-IG[a] v2.0	✓	
Paper mhGAP-IG[b] v2.0[c]	✓	✓
Remote supervision and support module	✓	✓
Primary care workers' training in use and administration of the relevant mhGAP-IG v2.0	✓	✓
Primary care workers' training in a clinical dashboard	✓	
Supervisors' training in relevant mhGAP-IG and remote supervision and support	✓	✓
Supervisors' training in a clinical dashboard	✓	

[a]e-mhGAP-IG: electronic Mental Health Gap Action Programme Intervention Guide.
[b]mhGAP-IG: Mental Health Gap Action Programme Intervention Guide.
[c]The paper mhGAP-IG v2.0 will be used in intervention condition training and will be available for use as a resource throughout the study.

Remote Supervision

In both study arms, remote supervision will comprise an initial face-to-face meeting (where possible) and subsequent contact by voice and text messaging to discuss implementation of the mhGAP-IG (paper or e-version) and any case queries. Supervision format (eg, voice calls, video calls, messaging, group, and individual) will be agreed upon by each supervision dyad. Supervisors will be provided with training on how to set up and run supervision remotely using telephone and other common communication platforms such as WhatsApp groups before the start of the study. Training will cover issues such as building rapport, format for running supervision over a telephone, format for clinically supportive WhatsApp groups, and ensuring patient confidentiality when using remote supervision methods. PCWs will receive training on how to make the most of the remote supervision during the mhGAP-IG training. PCWs will have access to project mobile phones to facilitate remote supervision. Both PCWs and supervisors will be provided with data packages.

Randomization and Allocation Concealment

Clinics will be randomized to the control or intervention conditions, with equal number of clinics in either group (1:1). Randomization will be carried out independently (to ensure concealment) by the trial statistician via a computer-generated random sequence before participants are recruited or the intervention is initiated. PCW selection will be performed according to health staffing levels before randomization of clinics to a study arm. Blinding or masking of participants, clinicians, and fieldworkers will not be possible, as it will be clear which conditions clinics or municipalities are assigned to during implementation and data collection. However, the senior and junior trial statisticians who carry out the randomization will not know the characteristics of the clinics being randomized, and the primary statistical analysis will also be blinded to allocation status.

Statistical Power and Sample Size Calculation

The study will take place in 1 government administrative region in Nepal and 4 in Nigeria. We will identify approximately 20 PHCs in Nepal and 6 in Nigeria. In each country, half of the PHCs will be randomly allocated to either the e-version or the paper version of the mhGAP-IG. As the clinics vary in size, we will assess at least two staff members in each clinic, with an estimated 2-5 health workers per facility and an overall enrollment of 40 PCWs in Nepal and 30 PCWs in Nigeria. Regarding patient numbers, on the basis of the sample size for the study by Sangha et al [34] on smartphone app for improving cognition to improve case detection, we will conduct within-arm comparison detection rates if at least 40 patients screen positive on the Patient Health Questionnaire (PHQ-9) in the research assistant interview in the 1-month pretraining period and in the first month of the posttraining patient enrollment period, which begins after 3 months of intensive supervision. This will allow us to detect a within-arm increase in the clinical case identification rate of 43% for the e-mhGAP-IG after the implementation of the e-version with 90% power at the 5% level of significance, assuming an intraclass correlation coefficient of 0.02. For example, with a 10% detection rate pretraining, 2 patients would receive a health worker depression diagnosis out of 20 patients screening positive. After training, the detection rate would increase to 53%, which equates to 11 patients diagnosed by a health worker out of 20 patients screening positive.

Data Collection

Overview

In line with the current best practice in implementation research [32], the research team will evaluate implementation processes and outcomes (eg, acceptability and feasibility) and factors that influence effectiveness implementation (eg, organizational readiness to change) across multiple stakeholder groups, including patients, PCWs, and supervisors, and at different stages of implementation [32,35]. This offers a 360-degree implementation evaluation, which considers the needs and perspectives that typically differ between stakeholder groups and can vary over time. Data collection procedures are outlined in Figure 1.

Implementation Data

PCWs Data

PCWs will be interviewed by a member of the research team during a 1-week period at each PHC clinic at 4 time points: (1) before mhGAP-IG training, (2) immediately post training, (3) 3 months post training, and (4) 8 months post training. During the research interviews, all PCWs will complete quantitative assessments, assessing 6 variables related to the implementation of the intervention:

1. Implementation readiness: Health workers' resolve and capability to implement the e-mhGAP-IG (ie, readiness to change) will be assessed using the Organizational Readiness for Implementing Change (ORIC) scale [36]. ORIC is a 12-item, theory-based measure assessing health workers' commitment toward and ability to implement change.

2. Acceptability, appropriateness, and feasibility: Acceptability, appropriateness, and feasibility of the mhGAP-IG and remote supervision will be assessed using the Acceptability of Intervention Measure (AIM), Intervention Appropriateness Measure (IAM), and Feasibility of Intervention Measure (FIM), respectively [37]. These brief, 4-item instruments have been developed by implementation scientists and mental health professionals and display good psychometric properties. Cultural and linguistic adaptation and further psychometric evaluation will be undertaken before the deployment of the measures in this study. The appropriateness, feasibility, and acceptability of the mhGAP-IG and remote supervision will also be assessed through qualitative interviews with health workers at the last data collection time point (PCW_3).

3. Fidelity: Patient records and app use, as recorded electronically through the e-mhGAP-IG v2.0, or patient records, as recorded on paper in clinics randomized to provide the paper version mhGAP-IG v2.0 and supervision notes, will be assessed by members of the research team to determine fidelity to the training manuals for the paper mhGAP-IG v2.0 and the adapted e-mhGAP-IG v2.0.

4. Adoption: Health workers' intention to adopt mhGAP-IG will be assessed by using 2 study-specific questions regarding provider-intended use within the study and after the study has ended. Intention to adopt at the provider level will also be assessed through qualitative interviews with health workers.

5. Integration and sustainability: The potential for long-term integration of the mhGAP-IG v2.0 within standard health services will be assessed using the NoMAD (Normalization Measure Development) scale [38,39]. NoMAD is a 23-item instrument that assesses staff perceptions of factors relevant to embedding interventions in health care. NoMAD consists of 4 theoretical constructs: (1) coherence, (2) collective action, (3) cognitive participation, and (4) reflexive monitoring.

6. Operating costs: The time taken to be trained and to use the paper and electronic tools and for remote supervision will be estimated from information collected from staff, and this, combined with information on staff wages, will be used to derive operating costs for the economic modeling.

During the last data collection time point (PCW_3), 15 PCWs in each country will be randomly selected and invited to participate in an additional qualitative interview with a member of the research team to gain further insight into their views on the implementation of the mhGAP-IG v2.0

and remote supervision. We will aim to conduct focus group discussions (FGDs) in the first instance. However, where this is not possible, individual interviews will be conducted. Individual interviews and FGDs will be audio recorded and last no longer than 30 and 60 minutes, respectively. The interviews will be transcribed and anonymized before analysis.

Supervisor Data

mhGAP-IG supervisors will be interviewed by a member of the research team during a 1-week period at 2 time points: (1) immediately post training and (2) 8 months post training (Figure 1). Interviews will focus on perceived acceptability, appropriateness, and feasibility of remote supervision using the quantitative assessments described above (ie, AIM, IAM, and FIM). All supervisors will be invited to participate in a qualitative interview 8 months post training to gain further insight into their experiences of remote supervision. FGDs will be conducted where possible, with the remaining selected participants completing individual interviews. All qualitative interviews will be audio recorded and last between 30 minutes for individual interviews and 60 minutes for FGDs. The interviews will be transcribed and anonymized before analysis.

Patient Data

We estimate screening a minimum of 400 patients at each site during the pretraining enrollment period and 1200 patients in the posttraining enrollment period (starting 3 months after the mhGAP-IG training). In the posttraining period, we will include a subset of patients who have received a health worker depression diagnosis to follow-up for treatment outcomes. We will follow up with them at approximately 3 months after they are screened to measure their treatment outcomes. A subset of patients will also participate in FGDs to assess their perspectives on the acceptability (AIM) and appropriateness (IAM) of the intervention 3 months after their baseline health care appointment.

Outcome Data

PCWs Data

PCWs will be interviewed by a member of the research team during a 1-week period at each PHC clinic at 4 time points: (1) before mhGAP-IG training, (2) immediately post training, (3) 3 months post training, and (4) 8 months post training. During the research interviews, all PCWs will complete quantitative assessments, assessing 5 variables related to the PCW outcomes:

1. mhGAP-IG Knowledge Scale: In the mhGAP-IG training, knowledge is assessed by a standardized set of 30 questions in the multiple-choice question format, the mhGAP-IG Knowledge Scale. These are administered before and after training to measure the change in knowledge.
2. Revised-Depression Attitude Questionnaire (R-DAQ): Previously used in both Nepal and Nigeria, the R-DAQ [40] assesses clinicians' views and understanding of depression. The R-DAQ 22-item scale asks clinicians to rate each item as *strongly disagree*, *disagree*, *neither disagree nor agree*, *agree*, or *strongly agree*. Examples of items include "depression is a disease like any other (eg, asthma, diabetes)," "psychological therapy tends to be

unsuccessful with people who are depressed," and "becoming depressed is a natural part of being old."

3. Social Distance Scale (SDS): The SDS was designed by Bogardus [41] to measure the level of acceptability of various types of social relationships between Americans and members of common ethnic groups [42,43]. The modified SDS has been widely used to measure mental health–related stigma and to understand the importance of labels attached to people with former mental illnesses [42,44]. The modified version consists of 12 items that represent social contact with different degrees of distance. The SDS measures the acceptability of different degrees of social distance and thus, by inference, the attitude of the respondent to the person with the condition [45]. The SDS sum score represents the attitude of the respondent toward the condition. The SDS has been used in global mental health research. Among stigma measures, it has been shown to be most strongly associated with health worker competence [46].

4. The Enhancing Assessment of Common Therapeutic Factors (ENACT)-clinician version: The ENACT-clinician version [47] is a measure of therapist competence that has been developed for use in training and supervision across settings varied by culture and access to mental health resources. A version adapted for mhGAP-IG trainings focusing on depression was used, with competencies rated on a 4-point scale from potentially harmful to done well, with good reliability (α=.89). Examples of items include "non-verbal communication, active listening," "assessment of functioning & impact on life," and "explanation and promotion of confidentiality," among others.

5. Perceptions of Supervisory Support Scale (PSS): Supervision quality will be assessed using the PSS [48]. The PSS is a 19-item scale that assesses perceived support. Subscales include emotional support, support for client goal achievement, and professional development support. Each item is rated using a 5-point Likert scale. Additional information about supervision quality will be collected through qualitative interviews with health workers at the last data collection time point.

Patient Data

In the month before mhGAP-IG training, the research team will collect depression diagnosis and treatment initiation data from patient baseline interviews and clinical notes at participating clinics. These data will be used as a baseline assessment of the accuracy of diagnosis and the adequacy of treatment initiation. Patients attending PHC 3-8 months following PCW mhGAP-IG training will be interviewed by a trained member of the research team either immediately before or after their clinic appointment. Quantitative measures will be used to assess depression, disability, intervention acceptability, and therapeutic alliance during individual interviews with a member of the research team as follows:

1. Depression: The PHQ-9 [49] is a self-administered diagnostic instrument for depressive disorders. The *Diagnostic and Statistical Manual of Mental Disorders, Fourth Edition* criteria for depression are scored as *0* (not at all) to *3* (nearly every day). A PHQ-9 score ≥10 had a sensitivity of 94% and 85% and a specificity of 80% and 99% for major depression in Nepal [50] and Nigeria [51], respectively.

2. Disability: Data on sociodemographic information (sex, age, education, marital status, and work status) will be collected through questions A1-A5 of the World Health Organization Disability Assessment Schedule 2.0 (WHODAS 2.0) [52]. The WHODAS 2.0 is a generic assessment instrument assessing health and disability across 6 domains (cognition, mobility, self-care, getting along, life activities, and participation). The research team will use the 12-item interviewer-administered version. The WHODAS 2.0 has been adapted and validated for use in Nepal [53-55] and Nigeria [56].

3. Treatment initiation: Treatment details will be extracted by the research team from patient records to document how diagnosis matches up with treatment and treatment modifications during care.

4. Therapeutic alliance: The ENACT-service user version [47] is a 15-item measure of patient experience of care and perception of therapeutic engagement. Example items include clear explanations, names for health problems, understanding and empathy, and expectations for recovery.

5. Suicidal ideation and behavior: Suicidal ideation and behavior will be assessed, in Nepal only, using suicidality questions adapted from the Composite International Diagnostic Interview suicidality module [57]. This tool has been widely used in Nepal [58]. We will ask participants whether they had thought of taking their own life in the past 12 months. Those who will respond affirmatively to the ideation question will be asked if they had made a plan to take their own life. In Nepal, those who meet the criteria for current suicidal thoughts or those who attempted suicide in the past 3 months will be immediately referred to a trained psychosocial counselor. In Nigeria, patients endorsing the suicidal ideation item from the PHQ-9 will receive further assessment of suicide risk in line with local protocols.

Confirmation of diagnosis and treatment initiation will be collected from the clinical notes by PCWs. Patients interviewed in the first 3 months of data collection and who screen positive for depression will be invited to participate in a follow-up interview 3 months after their first appointment. The interview will consist of quantitative assessments of depression (PHQ-9), disability (WHODAS 2.0), therapeutic alliance (the ENACT-service user version), and suicide ideation and action Composite International Diagnostic Interview. Treatment details will be extracted by the research team from patient records to document treatment retention.

Measure Translation and Adaptation

All standardized measures, except for the AIM, IAM, FIM, ORIC, NoMAD, and PSS (Nigeria only), have been translated and culturally adapted in Nepal and Nigeria. Psychometrics for translated and validated (when appropriate) measures are provided in the descriptions above. Within each site, where measures have not yet been adapted and validated, the International Test Commission Guidelines for Translating and Adapting Tests [59] will be followed, including cognitive interviewing [60,61] and the assessment of content validity.

Analysis

Implementation Outcome Analysis

Quantitative data will be assessed using generalized linear mixed models, depending on the distribution of the outcome (continuous, binary, and count). Descriptive statistics of the implementation survey data (FIM, AIM, and IAM) will be provided. The association between the primary outcome (changes in the PHQ-9 detection rate) and implementation survey data will be analyzed using linear mixed models at 3 and 8 months post training. A 2-level hierarchical model will be used, and all time points will be included as repeated measures in the model at baseline and at 3 months post training and 8 months post training to improve power and account for clustering of observations at patient and PCW levels. These models use maximum likelihood estimation and thus allow for missing outcome data under the missing at random assumption. Associations between secondary outcomes (eg, knowledge, attitudes, and competency) and implementation survey data will be assessed with a similar methodology for the primary outcomes, using generalized linear mixed models depending on the type of outcome (eg, normal, binary, and count). All analyses will be conducted using STATA V.15.1. (StataCorp).

Qualitative data will be assessed using thematic analyses [62]. Thematic analysis consists of 5 stages: familiarization, generating codes, constructing themes, revising themes, and defining themes [63]. Draft codebooks for each participant group (eg, PCWs, supervisors, and patients) will be developed on an initial subset of transcripts by 2 researchers at each site. Following refinement based on researcher consensus, the final codebooks will be used to code each transcript independently by 2 researchers at each site. Subcategories will be assessed for the number of occurrences across all transcripts and themes and categories relevant to the data identified. The findings will be triangulated with quantitative data on implementation and provider and patient outcomes to assess the feasibility and impact of the e-mhGAP-IG.

PCW and Patient Outcome Analysis

The primary outcome will be the accuracy of the depression diagnosis. The research team will identify a patient as screening positive based on the PHQ-9 result immediately before or after the patient sees the PCW. *Screening positive* will be defined as scoring ≥10 on the PHQ-9. The research team will also assess sensitivity to change, for change in both the PHQ-9 and WHODAS 2.0 scores from baseline and 3 months after initiation of treatment. *Positive diagnosis* will be defined as a clinical diagnosis of depression documented by the health worker in the clinical notes. *Accurate detection* will be defined as PHQ-9≥10 and a health worker depression clinical diagnosis. We will also report findings in terms of the sensitivity and specificity of health worker diagnoses. *Sensitivity* will be defined as the proportion of health worker–diagnosed patients who had PHQ-9 above the cutoff, out of all patients who scored above the PHQ-9 cutoff. *Specificity* will be defined as the proportion of patients who did not receive a health worker depression diagnosis and scored below the PHQ-9 cutoff, out of all patients scoring below the PHQ-9 cutoff.

Features of the cRCT design will be accommodated in all analyses, and an intention-to-treat approach will be used. The principles of analysis will be (1) the PHC-level accuracy outcome is binomial (ie, number of accurate diagnoses out of all diagnoses based on patient-level data), and therefore, a generalized linear mixed model will be used (specifically, a log-binomial regression to obtain probability ratios); (2) accuracy is a cumulative measure, and therefore, there are no repeated measures over time; and (3) missing data can occur at either the health worker or patient level.

Differences in detection rates will be assessed using patient outcome data at 3 months after enrollment, only in depression cases identified in each arm (ie, scoring ≥10 on the PHQ-9), and the health worker clinical diagnosis. A 3-level hierarchical model will be used when all time points will be included as repeated measures in the model to improve power and take into account clustering of the observation at patient and PHC levels. The 3-level linear mixed model will be used to estimate a 95% CI for the comparison of clinical diagnosis and PHQ-9 screening positive rates (as well as a subanalysis with PHQ-9 plus WHODAS 2.0 criteria) within electronic and paper mhGAP-IG versions. Secondary outcomes (eg, knowledge and attitudes) will be assessed within each arm with a similar methodology for the primary outcomes, using generalized linear mixed models depending on the type of outcome (normal, binary, and count). Clinical feasibility will also be triangulated using penetration and costing data.

Health Economic Analysis

We will develop a simulation model to assess the potential cost-effectiveness of the e-mhGAP-IG compared with the paper-based tool. We are not measuring patient outcomes within the study; hence, costs of services and impacts on patients will be taken from other sources. The model will take the form of a decision tree that maps out key events following the use of either tool and outcomes that are achieved. A simple form of the model will have cases detected or not as key events and whether or not outcomes improve as a result of detection (the latter information coming from previous research). The cost of providing care for people detected will be included as will the reduction in disability-adjusted life years (DALYs) following treatment. Some of the data will be obtained from within the study (costs of using the tools based on staff time and rate of detection of depression), whereas other data (costs of treatment and DALYs) will be obtained from the wider literature and expert opinion. The model will be subject to sensitivity analyses to address the uncertainty of the model parameters. The model will enable us to generate a cost per DALY avoided by using the tool.

Feasibility Criteria for Progression to Full Trial

The primary objective is to evaluate the feasibility and acceptability of the intervention, its implementation, and the trial procedures for the subsequent cRCT. We must establish indicators on what procedures to carry on to the full trial and where modifications should be made to study design or content. The overall feasibility and acceptability will be determined in the intervention arm by the following criteria at the end of the study to determine progression to the full trial:

- Identification of qualitative themes reporting that both PCWs and clients perceive group primary care mental health services as being acceptable, feasible, and appropriate
- Retention of at least 67% (27/40) of PCWs and patients through end line assessments
- Fewer than 15% (15/98) of missing items on outcome measures across all assessments or fewer than 15% (eg, 3/22 items on the R-DAQ) for each questionnaire with more than 10 items) or 50% (eg, 4/9 items on the PHQ-9; for each measure with 10 items or fewer) of missing items on an individual assessment.
- Presence of adverse events among fewer than 10% (4/40) of the participants and any serious adverse events.

In domains where criteria are met, we will retain the procedure for the full trial. In domains where criteria are not met, we will modify the procedures for the full trial, guided by data collected during interviews. The presence of any adverse events and/or serious adverse events will be addressed by the trial team to identify alternative strategies for the full trial and data safety monitoring committee. The number of feasibility and acceptability criteria that are not met will determine the extent of intervention and trial design modification. Ethical approval for this feasibility study was obtained from the following collaborating institutions:

- Psychiatry, Nursing, and Midwifery Research Ethics Committee, King's College London, United Kingdom (May 2020)
- University of Ibadan and University College Hospital Joint Ethics Committee, University of Ibadan, Nigeria (December 2018)
- Nepal Health Research Council for the Transcultural Psychosocial Organization Nepal (October 2020)
- WHO Ethics Review Committee, WHO, Geneva (May 2020).

Results

The Emilia project was funded in July 2018. Formative qualitative studies have been conducted in both countries, with results published from Nepal [64]. Data collection for the pilot feasibility cRCT began in January 2021 and is protected to be completed by March 2022. Activities have been delayed in both countries because of COVID-19.

Discussion

The mhGAP-IG enables greater access to evidence-based mental health care by targeting nonmental health specialists (eg, primary care doctors, nurses, and community health workers) as providers. Despite its use in more than 100 countries, a WHO consultation process identified the paper format to be a hindrance to its uptake, due to the burden on PCWs to carry the guide with them during appointments, and limited availability of mental health specialists to provide support and supervision. The Emilia project aims to address these barriers to scale. The existing WHO-developed electronic mhGAP-IG v2.0 has been adapted and refined for use in Nepal and Nigeria. Through a feasibility cRCT, its impact on detection and treatment initiation for depression, one of the most common mental conditions, will be tested along with stakeholders' perceptions of its implementation and suitability for scale. Although we believe that the availability of the e-mhGAP-IG will improve the demand and usability of the mhGAP-IG, the paper version will still play a vital role in settings where access to electronic technology and/or the internet is limited. The inclusion of a new remote supervision module for the ongoing support of health workers, made available in both trial arms, will allow us to assess its feasibility, appropriateness, and acceptability, in addition to its potential impact on the success of both the paper and electronic mhGAP-IG versions.

This feasibility trial will provide initial evidence of the utility and impact of the e-mhGAP-IG and remote supervision. The study takes advantage of the availability and potential of electronic technology and will advance work to reduce the mental health treatment gap around the world.

Acknowledgments

The Emilia program is a collaboration of research colleagues in 5 countries committed to improving the identification and treatment of mental health problems in LMICs. It is coordinated by the Centre for Global Mental Health, Institute of Psychiatry, Psychology, and Neuroscience at King's College London. This work was supported by the Medical Research Council (grant MR/S001255/1). NS, GT, and LH's research is supported by the National Institute for Health Research (NIHR) Applied Research Collaboration South London at King's College Hospital National Health Service (NHS) Foundation Trust. NS, GT, and LH are members of King's Improvement Science, which offers cofunding to the NIHR Applied Research Collaboration South London and comprises a specialist team of improvement scientists and senior researchers based at King's College London. GT's and HL's work was also supported by the Guy's and St Thomas' Charity for the On Trac project (EFT151101), and by the UK Medical Research Council (UKRI) in relation the Indigo Partnership (MR/R023697/1). HL is additionally supported by a grant by the National Axial Spondyloarthritis Society and the NIHR (RP-PG-0610-10066). NS and GT are further funded by the NIHR Global Health Research Unit on Health System Strengthening in sub-Saharan Africa, King's College London (GHRU 16/136/54), using aid from the UK government to support global health research. NS's research is further supported by the ASPIRES (Antibiotic Use Across Surgical Pathways-Investigating, Redesigning, and Evaluating Systems) research program, funded by the Economic and Social Research Council. TTS's research is supported by a UK Research and Innovation Future Leaders Fellowship (MR/T019662/1). The authors alone are responsible for the views expressed in this study, and they do not necessarily represent the views, decisions, or policies of the institutions with which they are affiliated or the funders.

ABBREVIATIONS

AIM

Acceptability of Intervention Measure

ASPIRES

Antibiotic Use Across Surgical Pathways-Investigating, Redesigning, and Evaluating Systems

cRCT

cluster randomized controlled trial

DALY

disability-adjusted life year

e-mhGAP-IG

electronic Mental Health Gap Action Programme Intervention Guide

Emilia

E-mhGAP Intervention Guide in Low- and Middle-Income Countries: Proof-of-Concept for Impact and Acceptability

ENACT

Enhancing Assessment of Common Therapeutic Factors

FGD

focus group discussion

FIM

Feasibility of Intervention Measure

IAM

Intervention Appropriateness Measure

LMIC

low- and middle-income country

mHealth

mobile health

mhGAP-IG

Mental Health Gap Action Programme Intervention Guide

NHS

National Health Service

NIHR

National Institute for Health Research

NoMAD

Normalization Measure Development

ORIC

Organizational Readiness for Implementing Change

PCW

primary care worker

PHC

primary health clinic

PHQ-9

Patient Health Questionnaire

PSS

Perceptions of Supervisory Support Scale

R-DAQ

Revised-Depression Attitude Questionnaire

SDS

Social Distance Scale

WHO

World Health Organization

WHODAS 2.0

World Health Organization Disability Assessment Schedule 2.0

Authors' Contributions: GT, TTS, IB, BAK, MJDJ, LH, OG, NPL, NS, and PMC were involved in designing the study. KC, NC, TD, HL, OG, NPL, LK, PP, and EPG adapted the intervention for electronic use. IB will oversee quantitative data analysis. HL will oversee qualitative data analysis. All authors will be involved in the interpretation of the results. TTS wrote the first draft of this manuscript. All authors have contributed to, read, and approved the final manuscript.

Conflicts of Interest: NS is the director of the London Safety and Training Solutions Ltd, which offers training in patient safety, implementation solutions, and human factors to health care organizations.

REFERENCES

1.Steel Z, Marnane C, Iranpour C, Chey T, Jackson JW, Patel V, Silove D. The global prevalence of common mental disorders: a systematic review and meta-analysis 1980-2013. Int J Epidemiol. 2014 Apr;43(2):476–93. doi: 10.1093/ije/dyu038. [DOI] [PMC free article] [PubMed] [Google Scholar]

2.GBD 2016 DALYs and HALE Collaborators Global, regional, and national disability-adjusted life-years (DALYs) for 333 diseases and injuries and healthy life expectancy (HALE) for 195 countries and territories, 1990-2016: a systematic analysis for the Global Burden of Disease Study 2016. Lancet. 2017 Sep 16;390(10100):1260–344. doi: 10.1016/S0140-6736(17)32130-X. https://linkinghub.elsevier.com/retrieve/pii/S0140-6736(17)32130-X. [DOI] [PMC free article] [PubMed] [Google Scholar]

3.Wang PS, Aguilar-Gaxiola S, Alonso J, Angermeyer MC, Borges G, Bromet EJ, Bruffaerts R, de GG, de GR, Gureje O, Haro JM, Karam EG, Kessler RC, Kovess V, Lane MC, Lee S, Levinson D, Ono Y, Petukhova M, Posada-Villa J, Seedat S, Wells JE. Use of mental health services for anxiety, mood, and substance disorders in 17 countries in the WHO world mental health surveys. Lancet. 2007 Sep 8;370(9590):841–50. doi: 10.1016/S0140-6736(07)61414-

7. http://europepmc.org/abstract/MED/17826169. [DOI] [PMC free article] [PubMed] [Google Scholar]

4.Thornicroft G, Chatterji S, Evans-Lacko S, Gruber M, Sampson N, Aguilar-Gaxiola S, Al-Hamzawi A, Alonso J, Andrade L, Borges G, Bruffaerts R, Bunting B, de Almeida JM, Florescu S, de Girolamo G, Gureje O, Haro JM, He Y, Hinkov H, Karam E, Kawakami N, Lee S, Navarro-Mateu F, Piazza M, Posada-Villa J, de Galvis YT, Kessler RC. Undertreatment of people with major depressive disorder in 21 countries. Br J Psychiatry. 2017 Dec;210(2):119–24. doi: 10.1192/bjp.bp.116.188078. doi: 10.1192/bjp.bp.116.188078. [DOI] [PMC free article] [PubMed] [Google Scholar]

5.Thornicroft G, Votruba N. Does the United Nations care about mental health? Lancet Psychiatry. 2016 Jul;3(7):599–600. doi: 10.1016/S2215-0366(16)30079-7. [DOI] [PubMed] [Google Scholar]

6.World Health Organization . Comprehensive Mental Health Action Plan 2013-2020. Geneva: World Health Organization; 2013. [Google Scholar]

7.Koschorke M, Padmavati R, Kumar S, Cohen A, Weiss HA, Chatterjee S, Pereira J, Naik S, John S, Dabholkar H, Balaji M, Chavan A, Varghese M, Thara R, Thornicroft G, Patel V. Experiences of stigma and discrimination of people with schizophrenia in India. Soc Sci Med. 2014 Dec;123:149–59. doi: 10.1016/j.socscimed.2014.10.035. https://linkinghub.elsevier.com/retrieve/pii/S0277-9536(14)00688-1. [DOI] [PMC free article] [PubMed] [Google Scholar]

8.Lasalvia A, Zoppei S, Van Bortel T, Bonetto C, Cristofalo D, Wahlbeck K, Bacle SV, Van Audenhove C, van Weeghel J, Reneses B, Germanavicius A, Economou M, Lanfredi M, Ando S, Sartorius N, Lopez-Ibor JJ, Thornicroft G, ASPEN/INDIGO Study Group Global pattern of experienced and anticipated discrimination reported by people with major depressive disorder: a cross-sectional survey. Lancet. 2013 Jan 05;381(9860):55–62. doi: 10.1016/S0140-6736(12)61379-8. [DOI] [PubMed] [Google Scholar]

9.Lempp H, Abayneh S, Gurung D, Kola L, Abdulmalik J, Evans-Lacko S, Semrau M, Alem A, Thornicroft G, Hanlon C. Service user and caregiver involvement in mental health system strengthening in low- and middle-income countries: a cross-country qualitative study. Epidemiol Psychiatr Sci. 2018 Feb;27(1):29–39. doi: 10.1017/S2045796017000634. http://europepmc.org/abstract/MED/29113598. [DOI] [PMC free article] [PubMed] [Google Scholar]

10.Schnyder N, Panczak R, Groth N, Schultze-Lutter F. Association between mental health-related stigma and active help-seeking: systematic review and meta-analysis. Br J Psychiatry. 2017 Apr;210(4):261–8. doi: 10.1192/bjp.bp.116.189464. [DOI] [PubMed] [Google Scholar]

11.Task shifting: rational redistribution of tasks among health workforce teams global recommendations and guidelines. Geneva: World Health Organization; 2007. pp. 1–88. [Google Scholar]

12.The mobile economy sub-Saharan Africa 2019. GSM Association. 2019. [2020-06-23]. https://www.gsma.com/mobileeconomy/wp-content/uploads/2020/03/GSMA_MobileEconomy2020_SSA_Eng.pdf.

13.Rijal P. Mobile subscriptions outnumber population. Kathmandu Post. 2016. Jun 14, [2020-07-14]. https://kathmandupost.com/money/2016/06/14/mobile-subscriptions-outnumber-population.

14.World Health Organization . Global diffusion of eHealth: making universal health coverage achievable, report of the third global survey on eHealth. Geneva: World Health Organization; 2016. pp. 1–156. [Google Scholar]

15.Divall P, Camosso-Stefinovic J, Baker R. The use of personal digital assistants in clinical decision making by health care professionals: a systematic review. Health Informatics J. 2013 Mar;19(1):16–28. doi: 10.1177/1460458212446761. [DOI] [PubMed] [Google Scholar]

16.World Health Organization . Recommendations on digital interventions for health system strengthening. Geneva: World Health Organaization; 2019. [PubMed] [Google Scholar]

17.World Health Organization . mhGAP Intervention Guide - Version 2.0 - for mental, neurological and substance use disorders in non-specialized health settings. Geneva: World Health Organization; 2019. pp. 1–173. [Google Scholar]

18.Dua T, Barbui C, Clark N, Fleischmann A, Poznyak V, van Ommeren M, Yasamy MT, Ayuso-Mateos JL, Birbeck GL, Drummond C, Freeman M, Giannakopoulos P, Levav I, Obot IS, Omigbodun O, Patel V, Phillips M, Prince M, Rahimi-Movaghar A, Rahman A, Sander JW, Saunders JB, Servili C, Rangaswamy T, Unützer J, Ventevogel P, Vijayakumar L, Thornicroft G, Saxena S. Evidence-based guidelines for mental, neurological, and substance use disorders in low- and middle-income countries: summary of WHO recommendations. PLoS Med. 2011 Nov;8(11):e1001122. doi: 10.1371/journal.pmed.1001122. https://dx.plos.org/10.1371/journal.pmed.1001122. [DOI] [PMC free article] [PubMed] [Google Scholar]

19.The World Bank - Nepal Country Profile. World Development Indicators database. [2020-06-23]. https://databank.worldbank.org/views/reports/reportwidget.aspx?Report_Name=CountryProfile&Id=b450fd57&tbar=y&dd=y&inf=n&zm=n&country=NPL.

20.Luitel NP, Baron EC, Kohrt BA, Komproe IH, Jordans MJ. Prevalence and correlates of depression and alcohol use disorder among adults attending primary health care services in Nepal: a cross sectional study. BMC Health Serv Res. 2018 Mar 27;18(1):215. doi: 10.1186/s12913-018-3034-9. https://bmchealthservres.biomedcentral.com/articles/10.1186/s12913-018-3034-9. [DOI] [PMC free article] [PubMed] [Google Scholar]

21.Luitel NP, Jordans MJ, Kohrt BA, Rathod SD, Komproe IH. Treatment gap and barriers for mental health care: a cross-sectional community survey in Nepal. PLoS One. 2017;12(8):e0183223. doi: 10.1371/journal.pone.0183223. https://dx.plos.org/10.1371/journal.pone.0183223. [DOI] [PMC free article] [PubMed] [Google Scholar]

22.Luitel NP, Jordans MJ, Adhikari A, Upadhaya N, Hanlon C, Lund C, Komproe IH. Mental health care in Nepal: current situation and challenges for development of a district mental health care plan. Confl Health. 2015;9:3. doi: 10.1186/s13031-014-0030-5. https://conflictandhealth.biomedcentral.com/articles/10.1186/s13031-014-0030-5. [DOI] [PMC free article] [PubMed] [Google Scholar]

23.Annual report 2073-74. Department of Health Services, Kathmandu, Nepal. 2017. [2021-06-01]. http://dohs.gov.np/wp-content/uploads/2018/04/Annual_Report_2073-74.pdf.

24.Chase LE, Marahatta K, Sidgel K, Shrestha S, Gautam K, Luitel NP, Dotel BR, Samuel R. Building back better? Taking stock of the post-earthquake mental health and psychosocial response in Nepal. Int J Ment Health Syst. 2018;12:44. doi: 10.1186/s13033-018-0221-3. https://ijmhs.biomedcentral.com/articles/10.1186/s13033-018-0221-3. [DOI] [PMC free article] [PubMed] [Google Scholar]

25.Nigeria Country Profile: The World Bank Group. World Development Indicators database. [2020-06-23]. https://databank.worldbank.org/views/reports/reportwidget.aspx?Report_Name=CountryProfile&Id=b450fd57&tbar=y&dd=y&inf=n&zm=n&country=NGA.

26.Adewuya AO, Atilola O, Ola BA, Coker OA, Zachariah MP, Olugbile O, Fasawe A, Idris O. Current prevalence, comorbidity and associated factors for symptoms of depression and generalised anxiety in the Lagos State Mental Health Survey (LSMHS), Nigeria. Compr Psychiatry. 2018;81:60–5. doi: 10.1016/j.comppsych.2017.11.010. [DOI] [PubMed] [Google Scholar]

27.Demyttenaere K, Bruffaerts R, Posada-Villa J, Gasquet I, Kovess V, Lepine JP, Angermeyer MC, Bernert S, de Girolamo G, Morosini P, Polidori G, Kikkawa T, Kawakami N, Ono Y, Takeshima T, Uda H, Karam EG, Fayyad JA, Karam AN, Mneimneh ZN, Medina-Mora ME, Borges G, Lara C, de Graaf R, Ormel J, Gureje O, Shen Y, Huang Y, Zhang M, Alonso J, Haro JM, Vilagut G, Bromet EJ, Gluzman S, Webb C, Kessler RC, Merikangas KR, Anthony JC, Von Korff MR, Wang PS, Brugha TS, Aguilar-Gaxiola S, Lee S, Heeringa S, Pennell B, Zaslavsky AM, Ustun TB, Chatterji S. Prevalence, severity, and unmet need for treatment of mental disorders in the World Health Organization World Mental Health Surveys. J Am Med Assoc. 2004;291(21):2581–90. doi: 10.1001/jama.291.21.2581. [DOI] [PubMed] [Google Scholar]

28.MIS Report: January 2019. Nepal Telecommunications Authority, Kathmandu, Nepal. [2021-06-01]. https://nta.gov.np/wp-content/uploads/Poush-MIS-2075.pdf.

29.Spotlight on Nigeria: Delivering a digital future London. GSM Association. 2018. [2020-06-23]. https://www.gsma.com/publicpolicy/wp-content/uploads/2019/02/GSMA-Spotlight-on-Nigeria-Report.pdf.

30.Khoja S, Scott R, Husyin N, Durrani H, Arif M, Faqiri F, Hedayat E, Yousufzai W. Impact of simple conventional and Telehealth solutions on improving mental health in Afghanistan. J Telemed Telecare. 2016 Dec;22(8):495–8. doi: 10.1177/1357633X16674631. [DOI] [PubMed] [Google Scholar]

31.Brown T, Wyatt J. Design thinking for social innovation. Develop Outreach. 2010;12(1):29–43. doi: 10.1596/1020-797X_12_1_29. doi: 10.1596/1020-797X_12_1_29. [DOI] [Google Scholar]

32.Proctor E, Silmere H, Raghavan R, Hovmand P, Aarons G, Bunger A, Griffey R, Hensley M. Outcomes for implementation research: conceptual distinctions, measurement challenges, and research agenda. Adm Policy Ment Health. 2011 Mar;38(2):65–76. doi: 10.1007/s10488-010-0319-

7. http://europepmc.org/abstract/MED/20957426. [DOI] [PMC free article] [PubMed] [Google Scholar]

33.Jordans MJ, Luitel NP, Kohrt BA, Rathod SD, Garman EC, De Silva M, Komproe IH, Patel V, Lund C. Community-, facility-, and individual-level outcomes of a district mental healthcare plan in a low-resource setting in Nepal: a population-based evaluation. PLoS Med. 2019;16(2):e1002748. doi: 10.1371/journal.pmed.1002748. https://dx.plos.org/10.1371/journal.pmed.1002748. [DOI] [PMC free article] [PubMed] [Google Scholar]

34.Sangha S, George J, Winthrop C, Panchal S. Confusion: delirium and dementia - a smartphone app to improve cognitive assessment. BMJ Qual Improv Rep. 2015;4(1):1592. doi: 10.1136/bmjquality.u202580.w1592. http://europepmc.org/abstract/MED/26732085. [DOI] [PMC free article] [PubMed] [Google Scholar]

35.Weiner BJ. A theory of organizational readiness for change. In: Nilsen P, Birken SA, editors. Handbook on Implementation Science. Cheltenham: Elgar Publishing; 2020. pp. 215–32. [Google Scholar]

36.Shea CM, Jacobs SR, Esserman DA, Bruce K, Weiner BJ. Organizational readiness for implementing change: a psychometric assessment of a new measure. Implement Sci. 2014;9:7. doi: 10.1186/1748-5908-9-7. http://www.implementationscience.com/content/9//7. [DOI] [PMC free article] [PubMed] [Google Scholar]

37.Weiner BJ, Lewis CC, Stanick C, Powell BJ, Dorsey CN, Clary AS, Boynton MH, Halko H. Psychometric assessment of three newly developed implementation outcome measures. Implement Sci. 2017;12(1):108. doi: 10.1186/s13012-017-0635-3. https://implementationscience.biomedcentral.com/articles/10.1186/s13012-017-0635-3. [DOI] [PMC free article] [PubMed] [Google Scholar]

38.Rapley T, Girling M, Mair FS, Murray E, Treweek S, McColl E, Steen IN, May CR, Finch TL. Improving the normalization of complex interventions: part 1 - development of the NoMAD instrument for assessing implementation work based on normalization process theory (NPT) BMC Med Res Methodol. 2018;18(1):133. doi: 10.1186/s12874-018-0590-y. https://bmcmedresmethodol.biomedcentral.com/articles/10.1186/s12874-018-0590-y. [DOI] [PMC free article] [PubMed] [Google Scholar]

39.Finch TL, Girling M, May CR, Mair FS, Murray E, Treweek S, McColl E, Steen IN, Cook C, Vernazza CR, Mackintosh N, Sharma S, Barbery G, Steele J, Rapley T. Improving the normalization of complex interventions: part 2 - validation of the NoMAD instrument for assessing implementation work based on normalization process theory (NPT) BMC Med Res Methodol. 2018;18(1):1–13. doi: 10.1186/s12874-018-0591-x. https://bmcmedresmethodol.biomedcentral.com/articles/10.1186/s12874-018-0591-x. [DOI] [PMC free article] [PubMed] [Google Scholar]

40.Haddad M, Menchetti M, McKeown E, Tylee A, Mann A. The development and psychometric properties of a measure of clinicians' attitudes to depression: the revised Depression Attitude Questionnaire (R-DAQ) BMC Psychiatry. 2015;15:7. doi: 10.1186/s12888-014-0381-

x. https://bmcpsychiatry.biomedcentral.com/articles/10.1186/s12888-014-0381-x. [DOI] [PMC free article] [PubMed] [Google Scholar]

41.Bogardus E. Measuring Social Distance. J Appl Sociol. 1925. [2021-06-01]. https://brocku.ca/MeadProject/Bogardus/Bogardus_1925c.html.

42.Link BG, Yang LH, Phelan JC, Collins PY. Measuring mental illness stigma. Schizophr Bull. 2004;30(3):511–41. doi: 10.1093/oxfordjournals.schbul.a007098. [DOI] [PubMed] [Google Scholar]

43.Parrillo V, Donoghue C. Updating the Bogardus social distance studies: a new national survey. Soc Sci J. 2005;42(2):257–71. doi: 10.1016/j.soscij.2005.03.011. doi: 10.1016/j.soscij.2005.03.011. [DOI] [Google Scholar]

44.Link B, Cullen F, Frank J, Wozniak J. The social rejection of former mental patients: understanding why labels matter. Am J Sociol. 1987;92(6):1461–500. doi: 10.1086/228672. doi: 10.1086/228672. [DOI] [Google Scholar]

45.Peters RM, Dadun. Van Brakel WH, Zweekhorst MB, Damayanti R, Bunders JF, Irwanto The cultural validation of two scales to assess social stigma in leprosy. PLoS Negl Trop Dis. 2014;8(11):e3274. doi: 10.1371/journal.pntd.0003274. https://dx.plos.org/10.1371/journal.pntd.0003274. [DOI] [PMC free article] [PubMed] [Google Scholar]

46.Kohrt BA, Mutamba BB, Luitel NP, Gwaikolo W, Mangen PO, Nakku J, Rose K, Cooper J, Jordans MJ, Baingana F. How competent are non-specialists trained to integrate mental health services in primary care? Global health perspectives from Uganda, Liberia, and Nepal. Int Rev Psychiatry. 2018;30(6):182–98. doi: 10.1080/09540261.2019.1566116. http://europepmc.org/abstract/MED/30810407. [DOI] [PMC free article] [PubMed] [Google Scholar]

47.Kohrt BA, Jordans MJ, Rai S, Shrestha P, Luitel NP, Ramaiya MK, Singla DR, Patel V. Therapist competence in global mental health: development of the ENhancing Assessment of Common Therapeutic factors (ENACT) rating scale. Behav Res Ther. 2015;69:11–21. doi: 10.1016/j.brat.2015.03.009. https://linkinghub.elsevier.com/retrieve/pii/S0005-7967(15)00048-0. [DOI] [PMC free article] [PubMed] [Google Scholar]

48.Fukui S, Rapp CA, Goscha R, Marty D, Ezell M. The perceptions of supervisory support scale. Adm Policy Ment Health. 2014;41(3):353–9. doi: 10.1007/s10488-013-0470-z. [DOI] [PubMed] [Google Scholar]

49.Kroenke K, Spitzer RL, Williams JB. The PHQ-9: validity of a brief depression severity measure. J Gen Intern Med. 2001 Sep;16(9):606–13. doi: 10.1046/j.1525-1497.2001.016009606.x. http://europepmc.org/abstract/MED/11556941. [DOI] [PMC free article] [PubMed] [Google Scholar]

50.Kohrt BA, Luitel NP, Acharya P, Jordans MJ. Detection of depression in low resource settings: validation of the Patient Health Questionnaire (PHQ-9) and cultural concepts of distress in Nepal. BMC Psychiatry. 2016;16:58. doi: 10.1186/s12888-016-0768-

y. https://bmcpsychiatry.biomedcentral.com/articles/10.1186/s12888-016-0768-y. [DOI] [PMC free article] [PubMed] [Google Scholar]

51.Adewuya AO, Ola BA, Afolabi OO. Validity of the patient health questionnaire (PHQ-9) as a screening tool for depression amongst Nigerian university students. J Affect Disord. 2006;96(1-2):89–93. doi: 10.1016/j.jad.2006.05.021. [DOI] [PubMed] [Google Scholar]

52.Üstün T, Kostanjsek N, Chatterji S, Rehm J, World Health Organization . In: Measuring health and disability: Manual for WHO disability assessment schedule WHODAS 2.0. Üstün TB, Kostanjsek N, Chatterji S, Rehm J, editors. Geneva: World Health Organization; 2010. [Google Scholar]

53.Tol WA, Kohrt BA, Jordans MJ, Thapa SB, Pettigrew J, Upadhaya N, de Jong JT. Political violence and mental health: a multi-disciplinary review of the literature on Nepal. Soc Sci Med. 2010;70(1):35–44. doi: 10.1016/j.socscimed.2009.09.037. [DOI] [PubMed] [Google Scholar]

54.Tol WA, Komproe IH, Jordans MJ, Thapa SB, Sharma B, De Jong JT. Brief multi-disciplinary treatment for torture survivors in Nepal: a naturalistic comparative study. Int J Soc Psychiatry. 2009;55(1):39–56. doi: 10.1177/0020764008091525. [DOI] [PubMed] [Google Scholar]

55.Thapa SB, Hauff E. Perceived needs, self-reported health and disability among displaced persons during an armed conflict in Nepal. Soc Psychiatry Psychiatr Epidemiol. 2012;47(4):589–95. doi: 10.1007/s00127-011-0359-7. http://europepmc.org/abstract/MED/21476014. [DOI] [PMC free article] [PubMed] [Google Scholar]

56.Adewuya AO, Ola BA, Coker OA, Atilola O, Zachariah MP, Olugbile O, Fasawe A, Idris O. Prevalence and associated factors for suicidal ideation in the Lagos State Mental Health Survey, Nigeria. BJPsych Open. 2016;2(6):385–9. doi: 10.1192/bjpo.bp.116.004333. https://www.cambridge.org/core/product/identifier/S2056472400001903/type/journal_article. [DOI] [PMC free article] [PubMed] [Google Scholar]

57.Robins LN, Wing J, Wittchen HU, Helzer JE, Babor TF, Burke J, Farmer A, Jablenski A, Pickens R, Regier DA. The composite international diagnostic interview. An epidemiologic instrument suitable for use in conjunction with different diagnostic systems and in different cultures. Arch Gen Psychiatry. 1988;45(12):1069–77. doi: 10.1001/archpsyc.1988.01800360017003. [DOI] [PubMed] [Google Scholar]

58.Aldridge LR, Garman EC, Luitel NP, Jordans MJ. Impact of a district mental health care plan on suicidality among patients with depression and alcohol use disorder in Nepal. PLoS One. 2020;15(4):e0231158. doi: 10.1371/journal.pone.0231158. https://dx.plos.org/10.1371/journal.pone.0231158. [DOI] [PMC free article] [PubMed] [Google Scholar]

59.Muñiz J, Elosua P, Hambleton RK, International Test Commission [International Test Commission Guidelines for test translation and adaptation: second edition] Psicothema. 2013;25(2):151–7. doi: 10.7334/psicothema2013.24. [DOI] [PubMed] [Google Scholar]

60.Paap MC, Lange L, van der Palen J, Bode C. Using the Three-Step Test Interview to understand how patients perceive the St. George's Respiratory Questionnaire for COPD patients (SGRQ-C) Qual Life Res. 2016;25(6):1561–70. doi: 10.1007/s11136-015-1192-

3. http://europepmc.org/abstract/MED/26615616. [DOI] [PMC free article] [PubMed] [Google Scholar]

61.Beatty P, Willis G. Research synthesis: the practice of cognitive interviewing. Public Opin Q. 2007;71(2):287–311. doi: 10.1093/poq/nfm006. doi: 10.1093/poq/nfm006. [DOI] [Google Scholar]

62.Braun V, Clarke V, Hayfield N, Terry G. Thematic analysis. In: Liamputtong P, editor. Handbook of Research Methods in Health Social Sciences. Singapore: Springer; 2018. pp. 978–81. [Google Scholar]

63.Braun V, Clarke V. Using thematic analysis in psychology. Qual Res Psychol. 2006;3(2):77–101. doi: 10.1191/1478088706qp063oa. doi: 10.1191/1478088706qp063oa. [DOI] [Google Scholar]

64.Pokhrel P, Karmacharya R, Taylor Salisbury T, Carswell K, Kohrt B A, Jordans M J D, Lempp H, Thornicroft G, Luitel N P. Perception of healthcare workers on mobile app-based clinical guideline for the detection and treatment of mental health problems in primary care: a qualitative study in Nepal. BMC Med Inform Decis Mak. 2021 Jan 19;21(1):21–93. doi: 10.1186/s12911-021-01386-0. https://bmcmedinformdecismak.biomedcentral.com/articles/10.1186/s12911-021-01386-0. [DOI] [PMC free article] [PubMed] [Google Scholar]
