# Supplementary material for: An App-Based WHO Mental Health Guide for Depression Detection: A Cluster Randomized Clinical Trial
Source: JAMA Netw Open. 2025 May 23;8(5):e2512064. doi: 10.1001/jamanetworkopen.2025.12064 (PMC12102703; doi:10.1001/jamanetworkopen.2025.12064)
Supplement: Supplement 2. — eMethods. Additional Information eFigure 1. Phases of the Feasibility Cluster Randomized Controlled Trial eFigure 2. Screenshots of e-mhGAP-IG App for Android Operating System eResults. Additional Information eFigure 3. Changes by Health Facility in Detection of Depression by Primary Care Workers in the Standard Mental Health Gap Action Programme–Intervention Guide Arm and Mobile App Version of the mhGAP-IG Arm eTable 1. Depression Detection by Facility and Country eFigure 4. App Usage by Country and Provider eFigure 5a. Median Scores With Interquartile Range for mhGAP Knowledge for Primary Care Providers eFigure 5b. Median Scores With Interquartile Range for Social Distance Scale (SDS) for Primary Care Providers eFigure 5c. Median Scores With Interquartile Range for Clinical Confidence (Revised Depression Attitudes Questionnaire, R-DAQ, Domain 1) for Primary Care Providers eFigure 5d. Median scores With Interquartile Range for Treatment Optimism (Revised Depression Attitudes Questionnaire, R-DAQ, Domain 2) for Primary Care Providers eFigure 5e. Median Scores With Interquartile Range for Perception of Depression (Revised Depression Attitudes Questionnaire, R-DAQ, Domain 3) for Primary Care Providers eFigure 5f. Median Scores With Interquartile Range for Helpful Behaviors on Clinical Competencies (Enhancing Assessment of Common Therapeutic Factors, ENACT) for Primary Care Providers eFigure 5g. Median Scores With Interquartile Range for Harmful Behaviors on Clinical Competencies (Enhancing Assessment of Common Therapeutic Factors, ENACT) for Primary Care Providers eTable 2. Means and 95% Confidence Intervals for Changes From Pre-Training to 8-Month Follow-Up Among Primary Care Providers‘ Knowledge, Attitudes, and Clinical Competence Adjusted for Clustering and Repeated Measures eReferences. [file jamanetwopen-e2512064-s002.pdf]

Supplementary Online Content

Kohrt BA, Ojagbemi A, Luitel NP, et al. An app-based WHO mental health guide for depression detection: a cluster randomized clinical trial. *JAMA Netw Open.* 2025;8(5):e2512064. doi:10.1001/jamanetworkopen.2025.12064

eMethods. Additional Information
eFigure 1. Phases of the Feasibility Cluster Randomized Controlled Trial
eFigure 2. Screenshots of e-mhGAP-IG App for Android Operating System
eResults. Additional Information
eFigure 3. Changes by Health Facility in Detection of Depression by Primary Care Workers in the Standard Mental Health Gap Action Programme–Intervention Guide arm and Mobile App version of the mhGAP-IG Arm
eTable 1. Depression Detection by Facility and Country
eFigure 4. App Usage by Country and Provider
eFigure 5a. Median Scores With Interquartile Range for mhGAP Knowledge for Primary Care Providers
eFigure 5b. Median Scores With Interquartile Range for Social Distance Scale (SDS) for Primary Care Providers
eFigure 5c. Median Scores With Interquartile Range for Clinical Confidence (Revised Depression Attitudes Questionnaire, R-DAQ, Domain 1) for Primary Care Providers
eFigure 5d. Median scores With Interquartile Range for Treatment Optimism (Revised Depression Attitudes Questionnaire, R-DAQ, Domain 2) for Primary Care Providers
eFigure 5e. Median Scores With Interquartile Range for Perception of Depression (Revised Depression Attitudes Questionnaire, R-DAQ, Domain 3) for Primary Care Providers
eFigure 5f. Median Scores With Interquartile Range for Helpful Behaviors on Clinical Competencies (Enhancing Assessment of Common Therapeutic Factors, ENACT) for Primary Care Providers
eFigure 5g. Median Scores With Interquartile Range for Harmful Behaviors on Clinical Competencies (Enhancing Assessment of Common Therapeutic Factors, ENACT) for Primary Care Providers
eTable 2. Means and 95% Confidence Intervals for Changes From Pre-Training to 8-Month Follow-Up Among Primary Care Providers‘ Knowledge, Attitudes, and Clinical Competence Adjusted for Clustering and Repeated Measures
eReferences.

This supplementary material has been provided by the authors to give readers additional information about their work.

eMethods. Additional Information

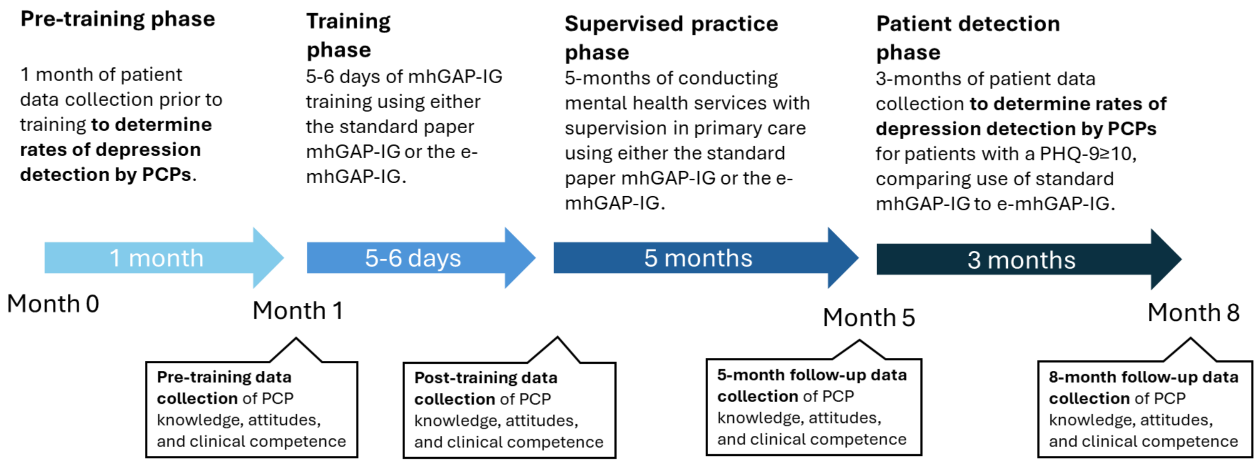

eFigure 1. Phases of the Feasibility Cluster Randomized Controlled Trial

Feasibility trial with standard mhGAP-IG arm and e-mhGAP-IG arm with the objective determine changes in rates of detection of depression by primary care providers from the pre-training phase prior to mhGAP-IG training compared to after training in the patient detection phase. (Abbreviations: mhGAP-IG: mental health Gap Action Programme-Intervention Guide; e-mhGAP: electronic mhGAP mobile app; PCP: primary care provider; PHQ-9: Patient Health Questionnaire)

Setting (additional information)

Nigeria and Nepal represent different LMIC contexts: Nepal has a gross national income per capita of $1,340, compared to $2,160 in Nigeria; literacy is 73% in Nepal and 78% in Nigeria. They also differ in culture and stages of healthcare development. Within Nepal, Jhapa is a southeastern district with a rural and semi-urban population of 1 million; approximately 66% of the national population lives in comparable rural or semi-urban areas. In Nigeria, Ibadan is the country's third largest city, with a population of 3.5 million; approximately 54% of the population lives in comparable urban areas.

© 2025 Kohrt BA et al. *JAMA Network Open.*

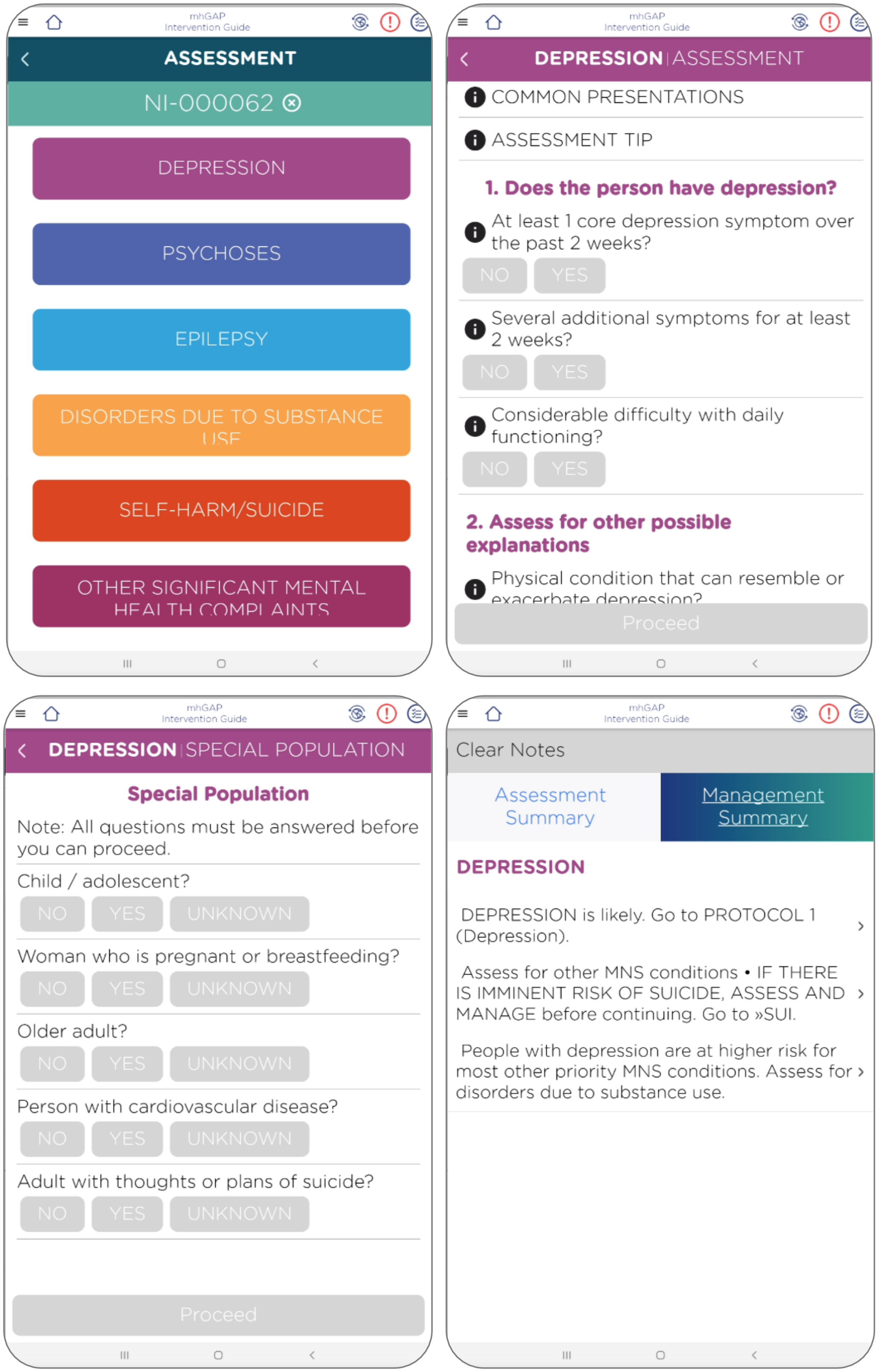

eFigure 2. Screenshots of e-mhGAP-IG App for Android Operating System

© 2025 Kohrt BA et al. *JAMA Network Open.*

Primary care provider tools

The mhGAP knowledge assessment tool included sections for depression and suicide.(World Health Organization, 2017) The Social Distance Scale (SDS)(Bogardus, 1925) was an adapted version with 12 items, which has previously been adapted for evaluating mhGAP trainings.(Kohrt et al., 2021; Kohrt et al., 2018) Higher scores on the SDS reflect greater desire for social distance and can be interpreted as higher levels of stigma. The Revised-Depression Attitudes Questionnaire (R-DAQ)(Haddad et al., 2015) includes 22 items, used to generate three domain scores: professional confidence in depression care; therapeutic optimism about depression; and general perspectives in depression.(Haddad et al., 2015) Higher scores on the R-DAQ represent more confidence and more positive attitudes towards providing care. Competency was evaluated with the WHO-UNICEF Ensuring Quality in Psychosocial and Mental Health Care (EQUIP) competency platform using standardized role plays, and the 15-item Enhancing Assessment of Common Therapeutic Factors (ENACT) tool.(Kohrt et al., 2015; Kohrt et al., 2024) At each of the assessment points, the PCPs participated in a 10-minute role play with an actor simulating a patient with depression. Scores are calculated for the number of harmful vs. the helpful clinical behaviors displayed in the role play. For each ENACT competency, there are approximately 3 harmful behavioral attributes (maximum=48 harmful behaviors) and approximately 5 helpful behavioral attributes (maximum=72 helpful behaviors).

Randomization and masking

Randomization was conducted at the level of the primary care facility. Research assistants administering the PHQ-9 were masked to the study arm of the facility. PCPs were blinded to the status of the PHQ-9 assessments of patients in their facilities.

Sample and statistical analysis

Identification of at least 40 patients with PHQ-9 score≥10 per arm, per country, was determined sufficient to evaluate with 90% power and a 5% level of significance, assuming an intraclass correlation coefficient (ICC) of 0.02.(Taylor Salisbury et al., 2021) This would enable detection of a within-arm increase in the depression detection rate of 43% for the e-mhGAP-IG (e.g., an increase from 10% depression detection in the pre-training phase to 53% in the post-training patient detection phase).

GEE analyses incorporated clustering within health facilities and repeated measures within individuals. Changes in detection rates were evaluated with linear mixed models for within arm comparisons between the 30-day pre-training phase and 90-day patient detection phase at 5-8 months post-training. Linear mixed models incorporated clustering of patients within facilities and repeated measures within facilities. This accounting for clustering within health facilities was the only adjustment made in the models. In Nigeria, because the pre-training phase detection rate was greater than zero, it was possible to also conduct GEE analyses to estimate the odds of detection after mhGAP-IG training compared to before mhGAP-IG training. Only PCPs with data available at

© 2025 Kohrt BA et al. *JAMA Network Open.*

the 8-month follow-up time point were included in the main PCP outcomes analyses comparing baseline (pre-training) vs. endline (8 months post-training), and no imputation was conducted for missing participants given the small sample size.

Health economic analyses

A costing template was completed for each country, including (i) number of training sessions, (ii) number and profession of trainers, (iii) length of sessions, (iv) number of attendees, (v) expenditure on trainer wages and training materials, and (vi) typical time taken to administer the tool including preparation and subsequent processing of information. Healthcare worker time spent using the tools was costed using estimated hourly wages, based on annual costs: Nepali Rupiya (NPR) 308,300 and Nigerian Naira (₦) 475,000. We assumed a working year consisted of 250 days and working day consisted of 7.5 hours. The hourly costs therefore are NPR164.43 for Nepal and ₦253.33 for Nigeria. Extra training costs were apportioned over the potential number of patients; we divided the costs by the number of people in the trial as a minimum estimate. We added the training costs per person to the tool administration costs per person and divided them by the study outcome. This was taken to be the extra proportion of people correctly identified as having depression.

Ethics, consent, and adverse events (additional information)

All PCPs and patients signed written consent forms. PCPs or patients who expressed psychological distress or who were determined by study staff to need support were referred to mental health services provided by the implementing organizations.

© 2025 Kohrt BA et al. *JAMA Network Open.*

eResults. Additional Information

Sample characteristics

In Nepal, there were 36 PCPs in the standard mhGAP-IG arm (8 female, 1 with prior mental health training) and 33 PCPs in the e-mhGAP-IG arm (7 female, 2 with prior mental health training). In Nepal, the median number of PCPs per facility was 3 for both arms. In Nigeria, there were 25 PCPs in the standard mhGAP-IG arm (24 female, 25 with prior mental health training) and 22 PCPs in the e-mhGAP-IG arm (20 female, 22 with prior mental health training). The median number of PCPs per facility in Nigeria was 6 in the standard mhGAP-IG arm and 5 in the e-mhGAP-IG arm.

Pre-training depression prevalence and detection rates

In Nepal, in the standard mhGAP-IG arm, 43 patients (17%) out of 246 scored above the PHQ-9 cut-off. In the e-mhGAP-IG arm, 49 patients (17%) out of 292 scored above the PHQ-9 cut-off. In Nigeria, in the standard mhGAP-IG arm, 36 patients (9%) out of 419 patients scored above the PHQ-9 cut-off. In the e-mhGAP-IG arm, 35 patients (8%) out of 446 score above the PHQ-9 cut-off. The ICC for detection of depression across all facilities was <0.1%.

Post-training depression prevalence and detection rates

At the follow-up period conducted from 5-8 months post PCP training, 92 patients (12%) out of 743 scored above the PHQ-9 cut-off the standard mhGAP-IG arm in Nepal. Of these, 15 (16%) were diagnosed by PCPs. In the e-mhGAP-IG arm, 91 (15%) out of 616 patients scored above the cut-off, of these 22 (24%) were diagnosed by PCPs. Of note, there was substantial variability across health facilities. One facility in the standard mhGAP-IG arm and two in the e-mhGAP-IG arm had no diagnoses among PHQ-9 cut-off patients. In the e-mhGAP-IG arm, there was one facility (Facility G3) where the majority of the diagnoses were made 18 out of 36 (50%). Of note, in Nepal, 6 patients were diagnosed with depression who scored below the PHQ-9 cut-off, i.e., 14% of the depression diagnoses made in Nepal were below the PHQ-9 score, this represents 0.5% of the 1,176 patients scoring below the cut-off receiving a PCP diagnosis of depression. Two of these diagnoses were in the standard mhGAP-IG arm and four in the e-mhGAP-IG arm.

In Nigeria, in the standard mhGAP-IG arm, 75 (8%) out of 917 patients scored above the cut-off, of whom 25 (33%) were diagnosed by PCPs. In the e-mhGAP arm, 76 out of 1077 (7%) patients scored above the cut-off, of whom 67 (88%) were diagnosed by PCPs. All facilities scored between 75-100% of patients above the PHQ-9 cut-off receiving a depression diagnosis. Of note, in Nigeria, none of the 1,843 patients who scored below a PHQ-9 cut-off were diagnosed with depression.

© 2025 Kohrt BA et al. *JAMA Network Open.*

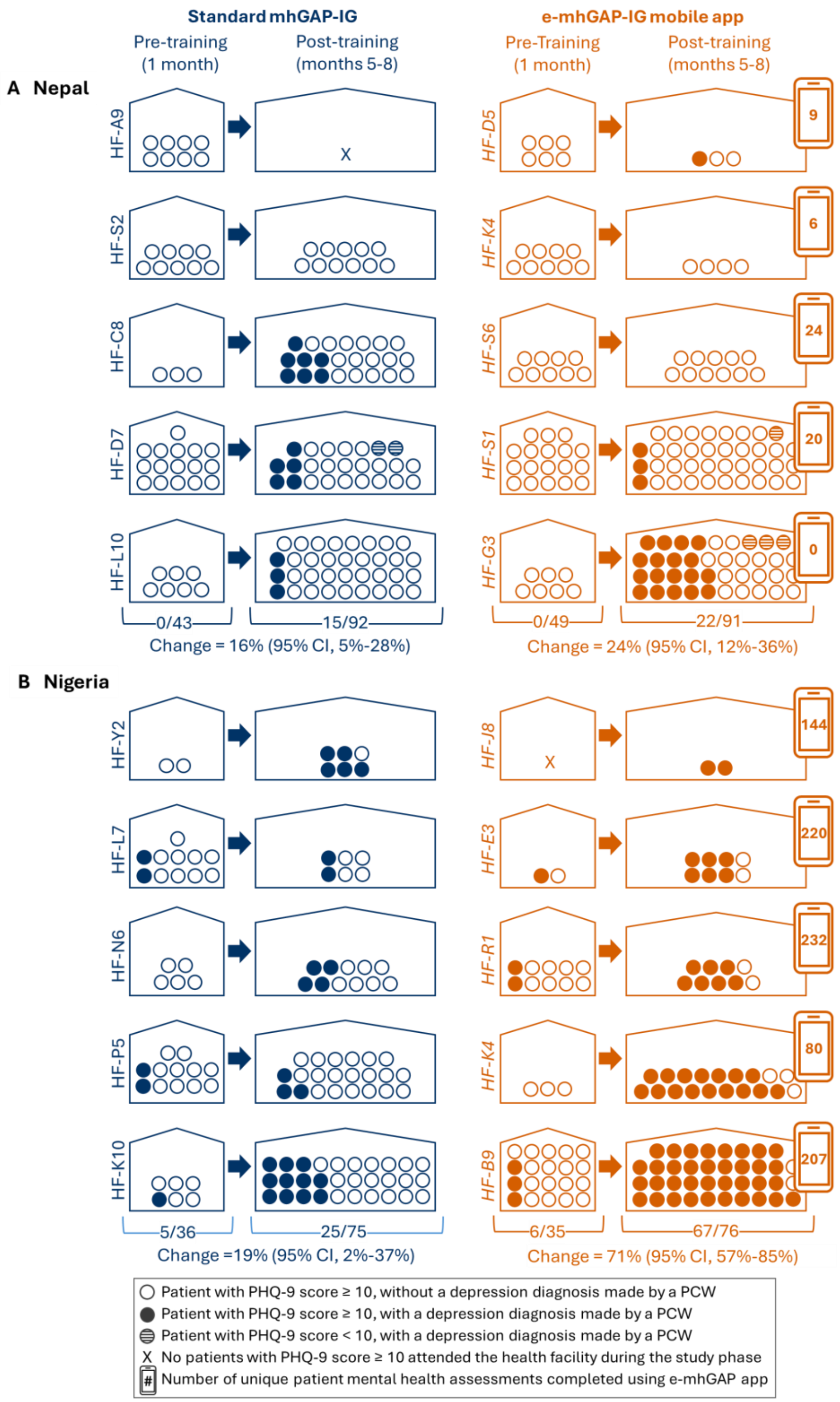

eFigure 3. Changes by Health Facility in Detection of Depression by Primary Care Workers (PCWs) in the Standard Mental Health Gap Action Programme–Intervention Guide (mhGAP-IG) arm and Mobile App version of the mhGAP-IG (e-mhGAP-IG) Arm in Nepal and Nigeria.

Number of patients with depression diagnosed by a PCWP are presented by health facility before training and during months 5 to 8 after training on the mhGAP-IG or the e-mhGAP-IG. Each circle represents 1 patient. The number of patients with whom the e-mhGAP-IG mobile app was used for assessment is depicted for each facility in the e-mhGAP-IG study arm in each country. In Nepal, a total of 616

patients presented to the facilities in the e-mhGAP-IG arm during months 5 to 8; in Nigeria, 1077 patients presented to facilities in the e-mhGAP-IG arm during this period. Pretraining to posttraining change and 95% CIs were calculated using linear mixed 12 models to account for clustering effects within health facilities. All 10 health facilities in Nigeria participated in the detection component of the study. In Nepal, 10 of 25 facilities participated in the detection component. HF indicates health facility code; and PHQ-9, 9-item Patient Health Questionnaire.

© 2025 Kohrt BA et al. *JAMA Network Open.*

eTable 1. Depression Detection by Facility and Country

Health Facility	Depression detection prior to PCP training			Depression detection during months 5-8 after PCP training		
	Patients with PHQ-9 ≥ 10	Depression diagnoses made by health workers	Percent detected by health workers	Patients with PHQ-9 ≥ 10	Depression diagnoses made by health workers	Percent detected by health workers
**Nepal**						
Standard mhGAP-IG						
A9	8	0	0%	0	0	--
S2	9	0	0%	11	0	0%
D7	16	0	0%	23	5	22%[A]
C8	3	0	0%	23	7	30%
L10	7	0	0%	35	3	9%
**Total**	**43**	**0**	**0%**	**92**	**15**	**16%**
e-mhGAP-IG						
D5	6	0	0%	3	1	33%
K4	9	0	0%	4	0	0%
S6	9	0	0%	11	0	0%
G3	7	0	0%	36	18	50%[A]
S1	18	0	0%	37	3	8%[A]
**Total**	**49**	**0**	**0%**	**91**	**22**	**24%**
**Nigeria**						
Standard mhGAP-IG						
Y2	2	0	0%	6	5	83%
L7	11	2	18%	6	2	33%
N6	5	0	0%	11	4	36%
P5	12	2	17%	22	3	14%
K10	6	1	17%	30	11	37%
**Total**	**36**	**5**	**14%**	**75**	**25**	**33%**
e-mhGAP-IG						
J8	0	0	--	2	2	100%
E3	2	1	50%	8	6	75%
R1	10	2	20%	9	7	78%
K4	3	0	0%	19	16	84%
B9	20	3	15%	38	36	95%
**Total**	**35**	**6**	**17%**	**76**	**67**	**88%**

[A] These facilities had additional patients diagnosed with depression; however, their PHQ-9 scores were below 10.

Abbreviations: e-mhGAP-IG, electronic mental health Gap Action Programme-Intervention Guide; mhGAP-IG, mental health Gap Action Programme-Intervention Guide; PHQ-9, Patient Health Questionnaire-9.

Usage of the e-mhGAP-IG mobile app

In Nepal, the median number of times the app was used per health facility for a patient assessment in Nepal was 9 (interquartile range, IQR: 3-22). In the 5 facilities in Nigeria, it was 207 (IQR: 112-226). The median number of times the app was used per PCP in Nepal was 0 (IQR: 0-6), and in Nigeria it was 32 (IQR: 18-48).

© 2025 Kohrt BA et al. *JAMA Network Open.*

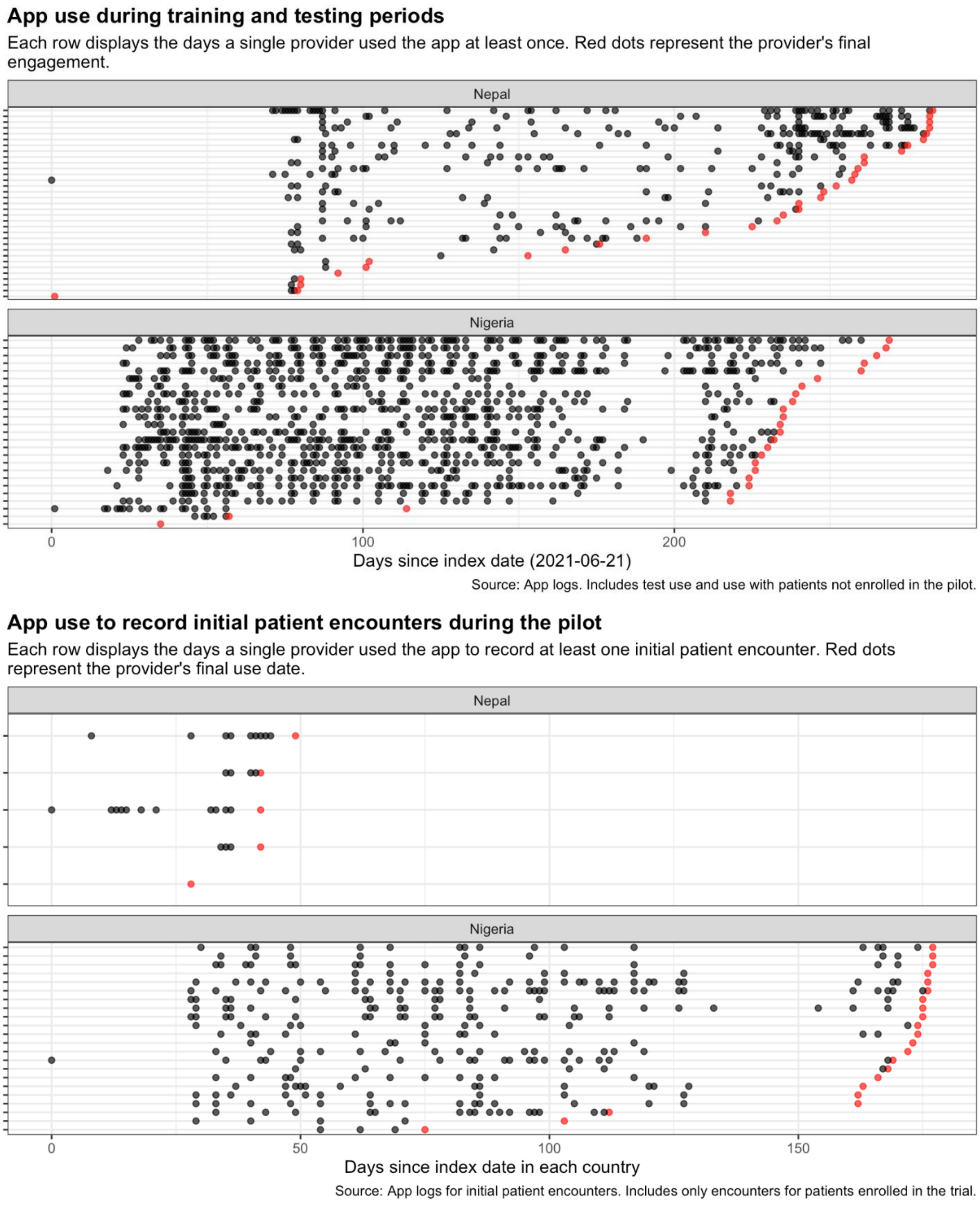

eFigure 4. App Usage by Country and Provider.

© 2025 Kohrt BA et al. *JAMA Network Open.*

Primary care providers' outcomes – intraclass correlation coefficients (ICCs)

In Nepal, the ICC for knowledge was 23%; in Nigeria, it was <0.1%. The SDS ICC at baseline in SDS was <0.1% in Nepal, and 8% in Nigeria. In Nepal, the ICC at baseline for the R-DAQ domain on professional confidence was 20%, the other two domains were <0.1%. In Nigeria, all ICCs for R-DAQ domains were <0.1%. In Nepal, helpful behaviors on the ENACT had an ICC of 27%, and the harmful behaviors were an ICC of <0.1%.

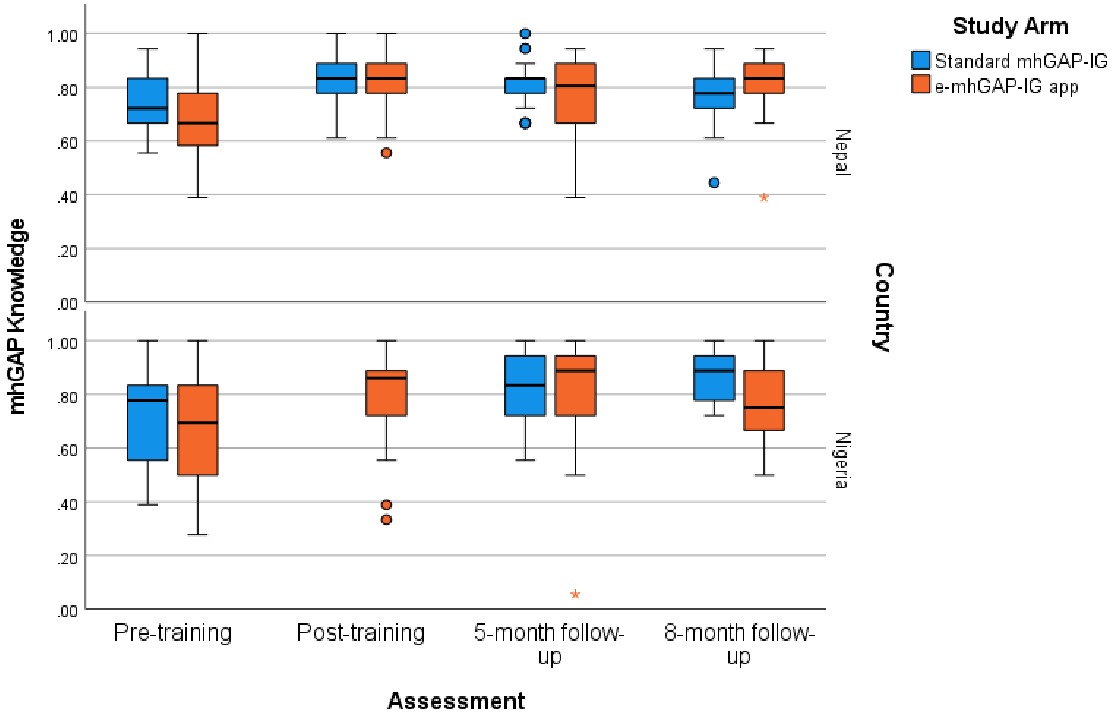

eFigure 5a. Median Scores With Interquartile Range for mhGAP Knowledge for Primary Care Providers

Sample size: Nepal, standard mhGAP-IG pre-training (n=36), post-training (n=36), 5-month follow-up (n=34), 8-month follow-up (n=33); e-mhGAP-IG pre-training (n=31), post-training (n=31), 5-month follow-up (n=26), 8-month follow-up (n=14); Nigeria, standard mhGAP-IG pre-training (n=25), post-training (n=0), 5-month follow-up (n=25), 8-month follow-up (n=25); e-mhGAP-IG pre-training (n=22), post-training (n=22), 5-month follow-up (n=22), 8-month follow-up (n=22).

© 2025 Kohrt BA et al. *JAMA Network Open.*

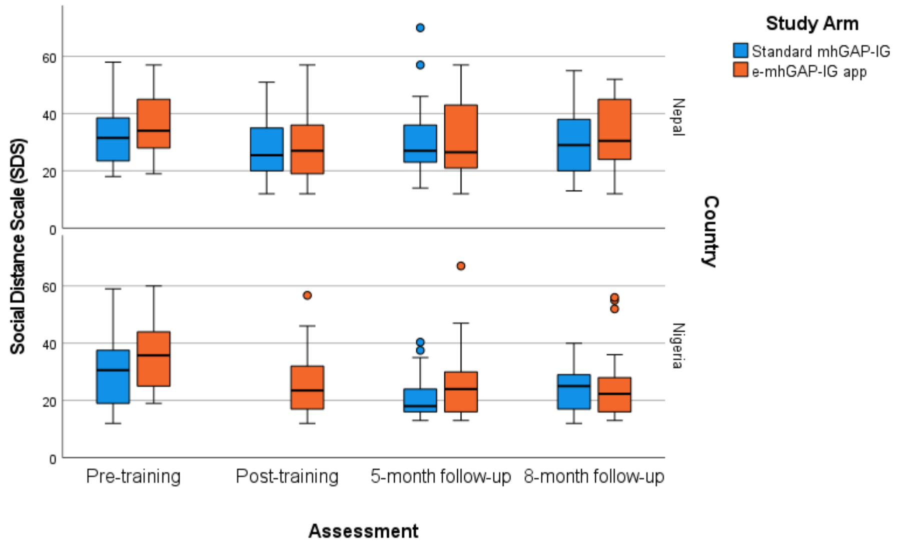

eFigure 5b. Median Scores With Interquartile Range for Social Distance Scale (SDS) for Primary Care Providers

Sample size: Nepal, standard mhGAP-IG pre-training (n=36), post-training (n=36), 5-month follow-up (n=34), 8-month follow-up (n=33); e-mhGAP-IG pre-training (n=31), post-training (n=31), 5-month follow-up (n=26), 8-month follow-up (n=14); Nigeria, standard mhGAP-IG pre-training (n=25), post-training (n=0), 5-month follow-up (n=25), 8-month follow-up (n=25); e-mhGAP-IG pre-training (n=22), post-training (n=22), 5-month follow-up (n=22), 8-month follow-up (n=22).

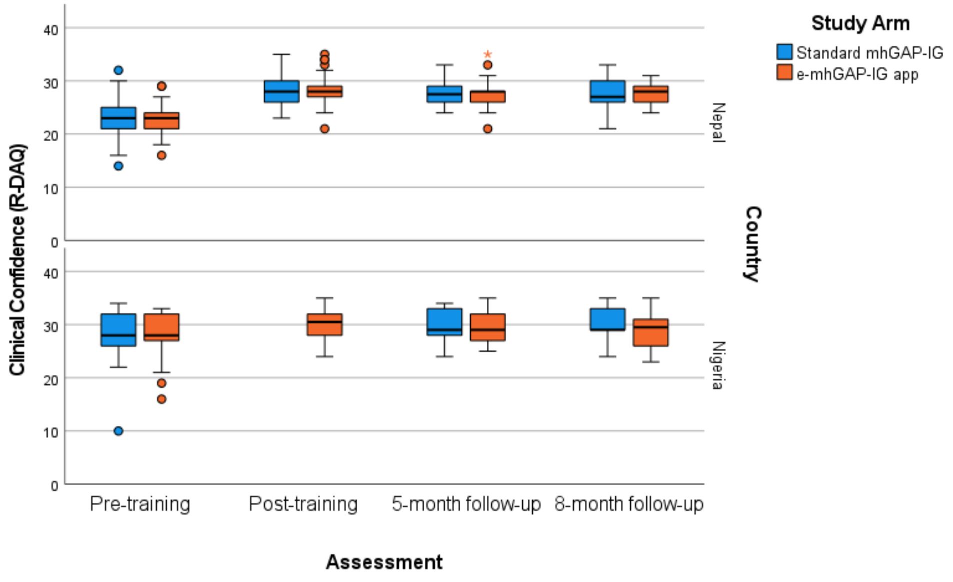

eFigure 5c. Median Scores With Interquartile Range for Clinical Confidence (Revised Depression Attitudes Questionnaire, R-DAQ, Domain 1) for Primary Care Providers

Sample size: Nepal, standard mhGAP-IG pre-training (n=36), post-training (n=36), 5-month follow-up (n=34), 8-month follow-up (n=33); e-mhGAP-IG pre-training (n=31), post-training (n=31), 5-month follow-up (n=26), 8-month follow-up (n=14); Nigeria, standard mhGAP-IG pre-training (n=25), post-training (n=0), 5-month follow-up (n=25), 8-month follow-up (n=25); e-mhGAP-IG pre-training (n=22), post-training (n=22), 5-month follow-up (n=22), 8-month follow-up (n=22).

© 2025 Kohrt BA et al. *JAMA Network Open.*

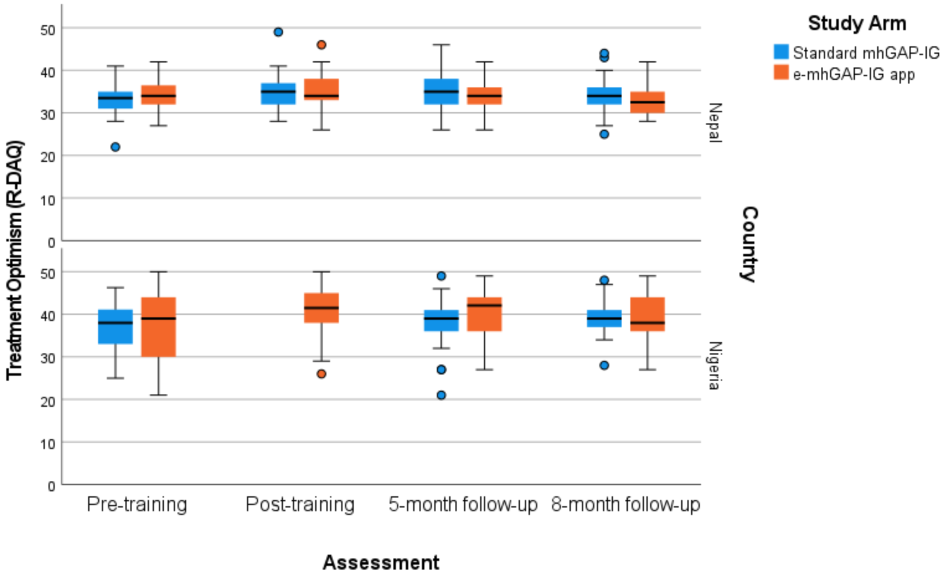

eFigure 5d. Median scores With Interquartile Range for Treatment Optimism (Revised Depression Attitudes Questionnaire, R-DAQ, Domain 2) for Primary Care Providers

Sample size: Nepal, standard mhGAP-IG pre-training (n=36), post-training (n=36), 5-month follow-up (n=34), 8-month follow-up (n=33); e-mhGAP-IG pre-training (n=31), post-training (n=31), 5-month follow-up (n=26), 8-month follow-up (n=14); Nigeria, standard mhGAP-IG pre-training (n=25), post-training (n=0), 5-month follow-up (n=25), 8-month follow-up (n=25); e-mhGAP-IG pre-training (n=22), post-training (n=22), 5-month follow-up (n=22), 8-month follow-up (n=22).

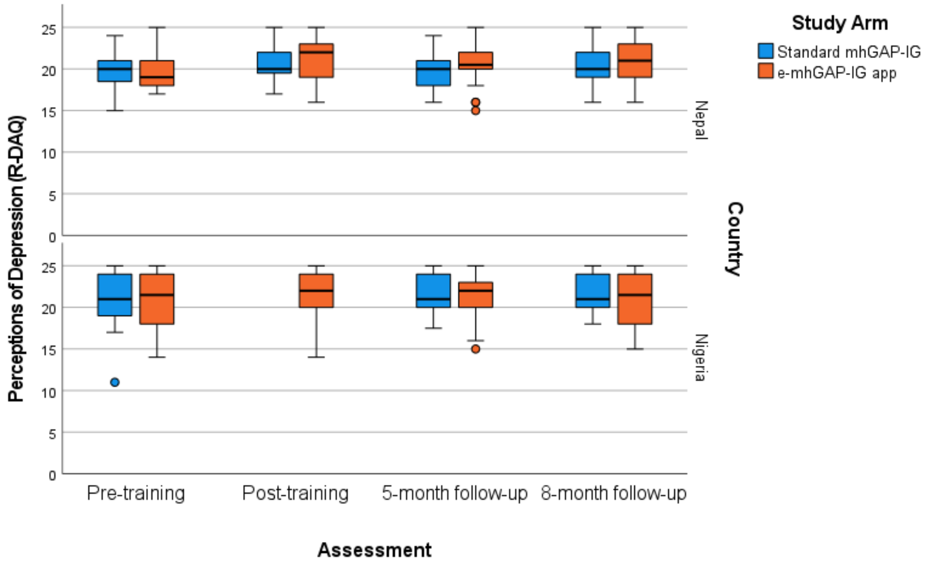

eFigure 5e. Median Scores With Interquartile Range for Perception of Depression (Revised Depression Attitudes Questionnaire, R-DAQ, Domain 3) for Primary Care Providers

Sample size: Nepal, standard mhGAP-IG pre-training (n=36), post-training (n=36), 5-month follow-up (n=34), 8-month follow-up (n=33); e-mhGAP-IG pre-training (n=31), post-training (n=31), 5-month follow-up (n=26), 8-month follow-up (n=14); Nigeria, standard mhGAP-IG pre-training (n=25), post-training (n=0), 5-month follow-up (n=25), 8-month follow-up (n=25); e-mhGAP-IG pre-training (n=22), post-training (n=22), 5-month follow-up (n=22), 8-month follow-up (n=22).

© 2025 Kohrt BA et al. *JAMA Network Open.*

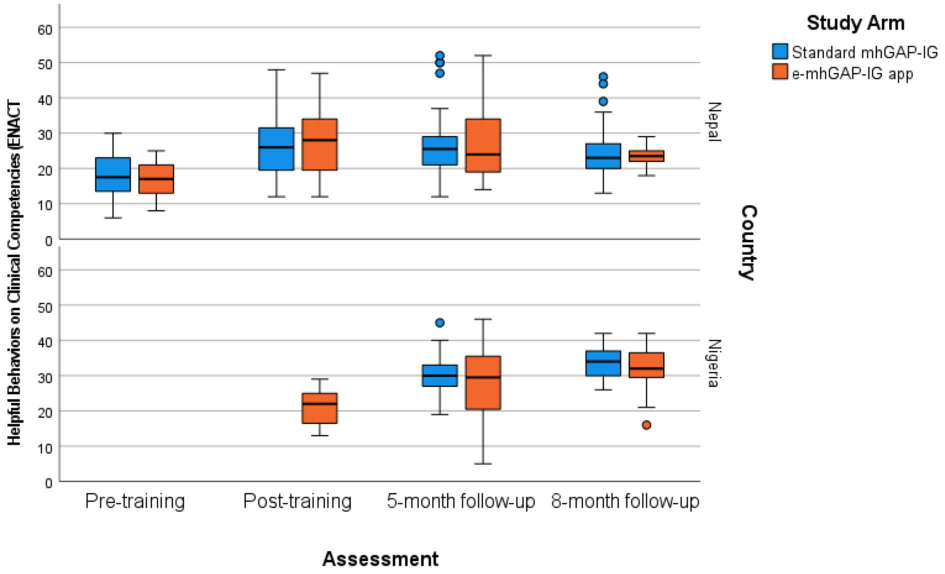

eFigure 5f. Median Scores With Interquartile Range for Helpful Behaviors on Clinical Competencies (Enhancing Assessment of Common Therapeutic Factors, ENACT) for Primary Care Providers

Sample size: Nepal, standard mhGAP-IG pre-training (n=36), post-training (n=36), 5-month follow-up (n=34), 8-month follow-up (n=33); e-mhGAP-IG pre-training (n=31), post-training (n=31), 5-month follow-up (n=26), 8-month follow-up (n=14); Nigeria, standard mhGAP-IG pre-training (n=0), post-training (n=0), 5-month follow-up (n=25), 8-month follow-up (n=25); e-mhGAP-IG pre-training (n=0), post-training (n=22), 5-month follow-up (n=22), 8-month follow-up (n=22).

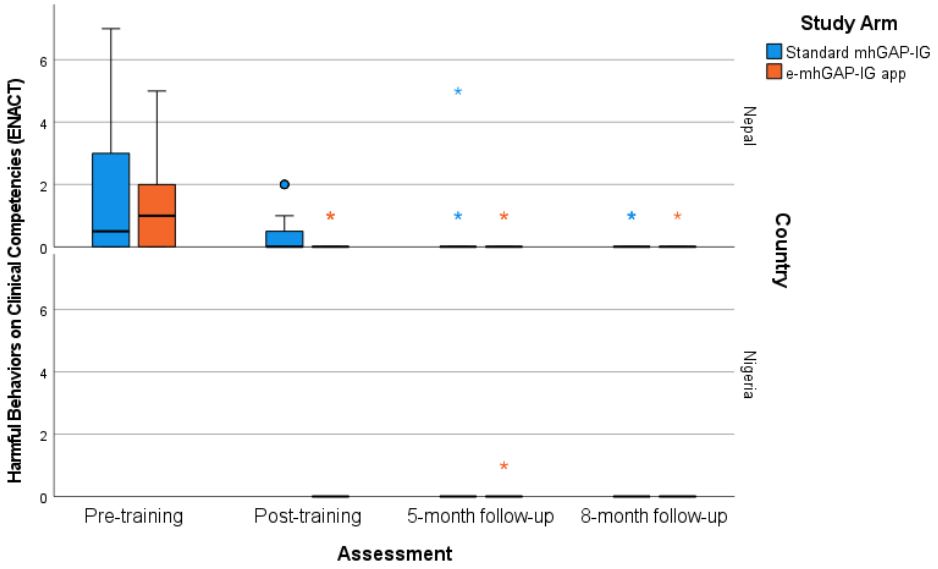

eFigure 5g. Median Scores With Interquartile Range for Harmful Behaviors on Clinical Competencies (Enhancing Assessment of Common Therapeutic Factors, ENACT) for Primary Care Providers

Sample size: Nepal, standard mhGAP-IG pre-training (n=36), post-training (n=36), 5-month follow-up (n=34), 8-month follow-up (n=33); e-mhGAP-IG pre-training (n=31), post-training (n=31), 5-month follow-up (n=26), 8-month follow-up (n=14); Nigeria, standard mhGAP-IG pre-training (n=0), post-training (n=0), 5-month follow-up (n=25), 8-month follow-up (n=25); e-mhGAP-IG pre-training (n=0), post-training (n=22), 5-month follow-up (n=22), 8-month follow-up (n=22).

© 2025 Kohrt BA et al. *JAMA Network Open.*

eTable 2. Means and 95% Confidence Intervals for Changes From Pre-Training to 8-Month Follow-Up Among Primary Care Providers' Knowledge, Attitudes, and Clinical Competence Adjusted for Clustering and Repeated Measures

	Nepal		Nigeria	
	Standard mhGAP-IG	e-mhGAP-IG app	Standard mhGAP-IG	e-mhGAP-IG app
**mhGAP Knowledge**	0.04<br>(0.01, 0.07)	0.14<br>(0.07, 0.20)	0.14<br>(0.07, 0.20)	0.08<br>(0.02, 0.15)
**Social Distance Scale (SDS)**	-3.48<br>(-7.36, 0.40)	-4.39<br>(-10.91, 2.13)	-6.60<br>(-11.10, -2.11)	-9.37<br>(-16.61, -2.13)
**Clinical Confidence (R-DAQ, domain 1)**	0.63<br>(0.49, 0.77)	0.66<br>(0.47, 0.84)	0.28<br>(-0.06, 0.62)	0.13<br>(-0.09, 0.35)
**Treatment Optimism (R-DAQ, domain 2)**	0.13<br>(-0.03, 0.30)	-0.13<br>(-0.38, 0.06)	0.21<br>(-0.07, 0.49)	0.18<br>(-0.16, 0.51)
**Perceptions of Depression (R-DAQ, domain 3)**	0.11<br>(-0.10, 0.23)	0.18<br>(-0.08, 0.43)	0.22<br>(-0.08, 0.51)	-0.06<br>(-0.41, 0.30)
**ENACT Helpful Behaviors**	7.38<br>(4.24, 10.31)	6.90<br>(5.23, 8.57)	--	--
**ENACT Harmful Behaviors**	-1.38<br>(-2.02, -0.74)	-1.19<br>(-1.70, -0.68)	--	--

Means and 95% confidence intervals are based on generalized estimating equations accounting for clustering within health facilities and repeated measures.

Economic evaluation (full details on estimation process)

In Nepal, the total training costs for both the e-mhGAP-IG and mhGAP-IG were Nepali Rupiya (NPR) 534,000 (USD 3,970). Dividing this by the 1,359 people in the post-training phase gives a cost per person of NPR 393. The administrative time was 30 minutes for the e-mhGAP-IG and 20 minutes for the mhGAP-IG, giving per person costs of NPR 82 NPR and NPR 55 respectively. Therefore, the total per person costs of e-mhGAP-IG are NPR 475 and for mhGAP-IG they are NPR 448. This represents an incremental cost of NPR 27 per person. The proportion of cases detected was 0.24 with e-mhGAP-IG giving a cost per case detected of NPR 1,980 (USD 14.79). The proportion was 0.16 for mhGAP-IG giving a cost per case detected of NPR 2,798 (USD 20.90). The incremental cost per extra person detected is NPR 343 (USD 2.54).

In Nigeria, total training costs for the e-mhGAP-IG were estimated at Naira (₦) 2,480,396 (USD 1,517). Dividing by the 1,994 patients in the trial for Nigeria at 3-month follow-up gives a cost per person of ₦1244. The training costs for the mhGAP-IG were ₦1,233,996 (USD 750) and the cost per person was ₦619. The administrative time was estimated to be 10 minutes for the e-mhGAP-IG and 3 minutes for the mhGAP-IG resulting in costs per person of ₦42 and ₦13 respectively. The total cost per person for e-mhGAP-IG was ₦1286 and for mhGAP-IG it was ₦632. Therefore, the incremental cost of the e-mhGAP-IG over the mhGAP-IG was ₦655. The proportion of cases detected was 0.88 with e-mhGAP-IG and 0.33 with mhGAP-IG, leading to costs of ₦1462 (USD 0.91) and ₦1,148 (USD 0.71) per case detected for each modality respectively. The incremental benefit in terms of detection was 0.55 (0.88-0.33) and so the incremental cost per extra person detected is ₦1,190 (USD 0.72).

© 2025 Kohrt BA et al. *JAMA Network Open.*

eReferences.

Bogardus, E. S. (1925). Measuring social distance. *Journal of applied sociology*, *9*, 299-308.

Haddad, M., Menchetti, M., McKeown, E., Tylee, A., & Mann, A. (2015). The development and psychometric properties of a measure of clinicians' attitudes to depression: the revised Depression Attitude Questionnaire (R-DAQ). *BMC Psychiatry*, *15*, 1-12.

Kohrt, B. A., Jordans, M. J. D., Rai, S., Shrestha, P., Luitel, N. P., Ramaiya, M., Singla, D., & Patel, V. (2015). Therapist Competence in Global Mental Health: Development of the Enhancing Assessment of Common Therapeutic Factors (ENACT) Rating Scale. *Behaviour Research and Therapy*, *69*, 11-21. https://doi.org/http://dx.doi.org/10.1016/j.brat.2015.03.009

Kohrt, B. A., Jordans, M. J. D., Turner, E. L., Rai, S., Gurung, D., Dhakal, M., Bhardwaj, A., Lamichhane, J., Singla, D. R., Lund, C., Patel, V., Luitel, N. P., & Sikkema, K. J. (2021). Collaboration with people with lived experience of mental illness to reduce stigma and improve primary care services in Nepal: a pilot cluster randomized clinical trial. *JAMA Network Open*, *4*(11), e2131475. https://doi.org/https://doi:10.1001/jamanetworkopen.2021.31475

Kohrt, B. A., Mutamba, B. B., Luitel, N. P., Gwaikolo, W., Onyango Mangen, P., Nakku, J., Rose, K., Cooper, J., Jordans, M. J. D., & Baingana, F. (2018). How competent are non-specialists trained to integrate mental health services in primary care? Global health perspectives from Uganda, Liberia, and Nepal. *Int Rev Psychiatry*, *30*(6), 182-198. https://doi.org/10.1080/09540261.2019.1566116

Kohrt, B. A., Pedersen, G. A., Schafer, A., Carswell, K., Rupp, F., Jordans, M. J. D., West, E., Akellot, J., Collins, P. Y., Contreras, C., Galea, J. T., Gebrekristos, F., Mathai, M., Metz, K., Morina, N., Mwenge, M. M., Steen, F., Willhoite, A., van Ommeren, M., . . . Yurtaev, A. (2024). Competency-based training and supervision: development of the WHO-UNICEF Ensuring Quality in Psychosocial and Mental Health Care (EQUIP) initiative. *The Lancet Psychiatry*. https://doi.org/https://doi.org/10.1016/S2215-0366(24)00183-4

Taylor Salisbury, T., Kohrt, B. A., Bakolis, I., Jordans, M. J., Hull, L., Luitel, N. P., McCrone, P., Sevdalis, N., Pokhrel, P., Carswell, K., Ojagbemi, A., Green, E. P., Chowdhary, N., Kola, L., Lempp, H., Dua, T., Milenova, M., Gureje, O., & Thornicroft, G. (2021). Adaptation of the World Health Organization Electronic Mental Health Gap Action Programme Intervention Guide App for Mobile Devices in Nepal and Nigeria: Protocol for a Feasibility Cluster Randomized Controlled Trial. *JMIR Res Protoc*, *10*(6), e24115. https://doi.org/10.2196/24115

World Health Organization. (2017). *mhGAP Training Manuals: for the mhGAP Intervention Guide for mental, neurological and substance use disorders in non-specialized health settings – version 2.0 (for field testing)*.

© 2025 Kohrt BA et al. *JAMA Network Open.*
