# Supplementary material for: An App-Based WHO Mental Health Guide for Depression Detection: A Cluster Randomized Clinical Trial
Source: JAMA Netw Open. 2025 May 23;8(5):e2512064. doi: 10.1001/jamanetworkopen.2025.12064 (PMC12102703; doi:10.1001/jamanetworkopen.2025.12064)
Supplement: Supplement 3. — Data Sharing Statement [file jamanetwopen-e2512064-s003.pdf]

Data Sharing Statement

Kohrt. An App-Based WHO Mental Health Guide for Depression Detection. *JAMA Netw Open*. Published May 23, 2025. doi:10.1001/jamanetworkopen.2025.12064

Data
Additional Information: Clinical trials.gov registration: NCT04522453
https://clinicaltrials.gov/ct2/show/NCT04522453
Data available: Yes
Data types: Deidentified participant data
How to access data: Principal Investigator: Graham Thornicroft graham.thornicroft@kcl.ac.uk
Statistics Lead: Ioannis Bakolis ioannis.bakolis@kcl.ac.uk
When available: With publication

Supporting Documents
Document types: None

Additional Information
Who can access the data: Individual de-identified participant data are available by application to the principal investigator (GT) and/or statistics lead (IB) from bona fide researchers interested in undertaking meta-analyses or on-going research, ordinarily with one or more of the original study PIs as collaborator or sponsor, in line with our institutional policies, from the date of publication for five years. Data files shared in this way may not then be shared with others.
Types of analyses: Meta-analyses and exploratory analyses.
Mechanisms of data availability: With investigator support after approval of the proposal by the PI (GT) or study statistician (IB).
